# Microstructure is associated with motor outcomes following Deep Brain Stimulation in Parkinson’s disease

**DOI:** 10.1038/s41531-025-00930-3

**Published:** 2025-04-21

**Authors:** Philipp Alexander Loehrer, Julia Freigang, Miriam H. A. Bopp, Alexander Calvano, Haidar S. Dafsari, Julius Wichmann, Amelie Seidel, Carolin Aberle, Susanne Knake, Christopher Nimsky, Lars Timmermann, Marcus Belke, David J. Pedrosa

**Affiliations:** 1 Department of Neurology, Philipps-University Marburg, Marburg, Germany; 2 MRC Brain Network Dynamics Unit, Nuffield Department of Clinical Neurosciences, University of Oxford, Oxford, UK; 3 Department of Neurosurgery, Philipps-University Marburg, Marburg, Germany; 4 Center for Mind, Brain and Behavior (CMBB), Marburg, Germany; 5 Department of Neurology, University Hospital Cologne, Cologne, Germany; 6 Center for Personalized Translational Epilepsy Research (CePTER) Consortium, Marburg, Germany

**Keywords:** Medical research, Parkinson's disease, Computational neuroscience, Predictive markers

## Abstract

Deep brain stimulation of the subthalamic nucleus (STN-DBS) is an established intervention for alleviating both motor and non-motor symptoms in advanced Parkinson’s disease (PD). However, patient outcomes may vary widely, underscoring the need for predictive biomarkers. Neuroimaging techniques, such as neurite orientation dispersion and density imaging (NODDI), a biophysical model-based MRI technique, offer promise in forecasting clinical outcomes and supporting preoperative counseling. This prospective, open-label study aimed to identify microstructural markers that correlate with short-term motor outcomes following STN-DBS in PD patients. Thirty-five patients underwent diffusion MRI and comprehensive clinical evaluations preoperatively and six months postoperatively. Evaluations were performed in the ON-medication as well as ON-medication/ON-stimulation state. A whole-brain voxel-wise analysis was conducted to explore associations between microstructural metrics and motor outcomes. Permutation-based statistical methods were applied to adjust for multiple comparisons. Intact microstructure in the bilateral putamen, bilateral insula, and left pallidum was significantly associated with a greater postoperative motor symptom improvement. Additionally, preserved microstructure in the pre- and postcentral gyrus and right precuneus was associated with increased duration with good mobility and without troublesome dyskinesia, and reduced time with poor mobility. These findings suggest that diffusion MRI may serve as valuable tool for identifying patients likely to exhibit favorable motor outcomes following STN-DBS. Incorporating microstructural data into preoperative counseling could enhance patient selection and optimize therapeutic strategies.

## Introduction

Deep brain stimulation (DBS) of the subthalamic nucleus represents an effective intervention for both motor and non-motor symptoms in Parkinson’s disease (PD)^1–5^. In addition to its local effects at the micro- and mesoscale, it is posited that the efficacy of DBS is derived from its ability to modulate distributed neurophysiological networks^6,7^. Consequently, the microstructural integrity of the brain regions constituting these networks is believed to be crucial for the therapeutic effectiveness of DBS^8^. Recent findings have demonstrated that the favorable outcomes related to non-motor symptoms are contingent upon the preservation of microstructural integrity within specific areas of the basal-ganglia-thalamo-cortical circuit^8^. These outcomes, along with evidence that preoperative levodopa response, tremor-dominant phenotype, baseline frontal score, and off-medication UPDRS part III scores predict short-term motor outcomes^9^, lend credence to the notion that quantitative MRI techniques, such as neurite orientation dispersion and density imaging (NODDI), may serve a valuable role in preoperative counseling^8,10^ - a concept that has gained increasing attention in recent years^11^. NODDI is a sophisticated, multi-compartmental diffusion-weighted MRI technique, employed to investigate the microstructure of brain tissue^12^. This model yields two voxelwise metrics pertaining to neurite morphology: the neurite density index (NDI), which quantifies the density of axons and dendrites within a voxel, and the orientation dispersion index (ODI), which assesses the degree of neurite dispersion, reflecting their alignment and dendritic arborization^12^. Importantly, the relationship between NODDI metrics and the underlying tissue properties has recently been corroborated through histological analysis^13,14^. Furthermore, NODDI models have been demonstrated to possess higher sensitivity compared to conventional DTI models, highlighting their potential utility as clinical tools^11^. Additionally, NODDI as a supplementary DTI technique exhibits robustness to variations in acquisition parameters with clinically feasible data acquisition times^11^.

Previous investigations utilizing NODDI in PD have illustrated the model’s capacity to identify disease-related pathology, such as retrograde degeneration of the nigrostriatal pathway^11^. Furthermore, NODDI metrics have been associated with bimanual motor control, disease severity, and disease duration^11,15^. The integration of NODDI with conventional DTI metrics in a multi-parametric analysis thus provides complementary insights into microstructural properties and may serve as an imaging-based biomarker for predicting treatment outcomes. Consequently, the present study aims to demonstrate that this approach can discern microstructural properties within brain regions that are associated with motor outcomes after STN-DBS in PD. Specifically, the study concentrates on identifying regions where microstructural metrics correlate with (1) alterations in the overall burden of motor symptoms, as assessed by the Unified Parkinson’s Disease Rating Scale (UPDRS), and (2) variations in periods characterized by good mobility and without troublesome dyskinesia (ON) versus these marked by poor mobility (OFF). The findings of this study could facilitate preoperative patient counseling by identifying specific brain microstructure that predict above- or below-average motor responses to STN-DBS.

## Results

### Clinical outcomes

**Fig. 1:**
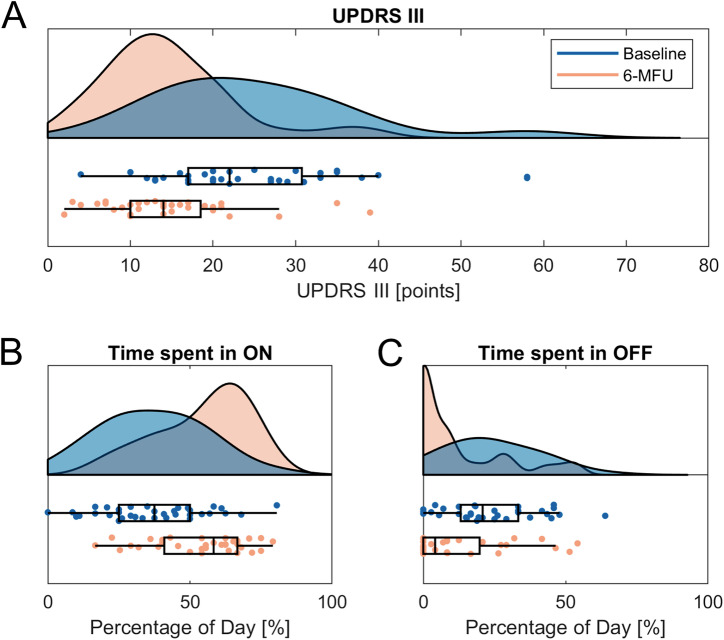
Visualization of baseline and 6-month follow-up values of the UPDRS part III (**A**), time spent with good mobility and no troublesome dyskinesia (ON, **B**), and time spent with poor mobility (OFF, **C**). Center line indicates the median, box limits represent upper and lower quartiles and whiskers indicate most extreme data points not considered outliers.

Thirty-five patients with PD (26 males, mean age 58.7 ± 7.4 years) were analyzed. At the six-month follow-up, the following primary outcomes improved: Unified Parkinson’s Disease Rating Scale (UPDRS) part III (*p* < 0.001, Cohen’s d = 1.02), duration with good mobility and without troublesome dyskinesia (ON, *p* < 0.001, Cohen’s d = −0.99), and duration with poor mobility (OFF, *p* < 0.001, Cohen’s d = 0.75). Furthermore, the following scales improved: UPDRS total score (*p* < 0.001, Cohen’s d = 0.94), UPDRS part I (*p* = 0.011, Cohen’s d = 0.48), UPDRS part IV (*p* < 0.001, Cohen’s d = 0.76), Parkinson’s Disease Questionnaire (PDQ)-8 summary index (SI) (*p* < 0.001, Cohen’s d = 0.69), Levodopa equivalent daily dose (LEDD; *p* < 0.001, Cohen’s d = 1.3), and LEDD of dopamine agonists (*p* < 0.001, Cohen’s d = 0.7). Analysis of movement records additionally revealed a reduction of duration with good mobility and troublesome dyskinesia (ON TD) after STN-DBS (*p* = 0.001, Cohen’s d = 0.55). Longitudinal changes of clinical outcomes are reported in Table 1 and Fig. 1. Longitudinal changes in clinical outcomes of the three- and twelve-month follow-up visits are reported in Supplementary Table 6 and demonstrate consistent effects with those reported above.

**Table 1:** Baseline characteristics and outcomes at baseline and 6-month follow-up

	*n*	*M*	*SD*					
Age [y]	35	58.7	7.4					
Disease duration [y]	35	8.9	4.5					
Sex (female/male) [%]	35	9/26	[25.7/74.3]					
	**Baseline**			**6-MFU**			**Baseline vs**. **6-MFU**	
	*n*	*M*	*SD*	*n*	*M*	*SD*	*p*	*Cohen’s d*
UPDRS total score	34	40.7	14.7	35	28.3	11.7	**<0.001**	**0.94**
UPDRS part I	34	2.9	2.1	35	1.9	1.8	**0.011**	**0.48**
UPDRS part II	34	8.3	5.4	35	8.1	5.3	0.562	0.04
UPDRS part III	35	24.9	11.9	35	14.5	8.0	**<0.001**	**1.02**
UPDRS part IV	34	6.0	3.1	35	3.8	2.6	**<0.001**	**0.76**
ON [%]	35	37.1	18.6	35	54.5	16.4	**<0.001**	−**0.99**
OFF [%]	35	23.9	15.9	35	11.8	16.4	**<0001**	**0.75**
SLEEP [%]	35	31.9	11.1	35	32.0	7.9	0.97	−0.007
ON TD [%]	35	7.0	12.65	35	1.7	5.6	**0.001**	**0.55**
PDQ-8 SI	35	32.8	15.3	35	22.6	14.4	**<0.001**	**0.69**
LEDD [mg]	35	964.9	408.3	35	531.2	267.5	**<0.001**	**1.3**
LEDD DA [mg]	35	266.5	130.7	35	176.9	125.3	**<0.001**	**0.7**

Demographic characteristics and outcome parameters at baseline and 6-months follow-up. Values for movement records represent percentage values. Reported p-values are corrected for multiple comparisons using Benjamini-Hochberg’s method for eight scales. Bold font highlights significant results, *p* < .05.

### Interaction between fractional anisotropy and postoperative change in motor symptoms

We evaluated the relationship between DTI metrics and postoperative motor symptom change using a generalized linear model corrected for multiple comparisons using a permutation-based approach. Lower regional mean fractional anisotropy values in the anterior division of the right cingulate gyrus, the right inferior fronto-occipital fasciculus, and the left superior longitudinal fasciculus were significantly associated with greater postoperative change in motor symptom burden, as indicated by changes in the UPDRS part III values (negative clusters N1, N2, and N7 cluster-wise p-value (CWP): <0.001–0.035). No clusters demonstrating a positive association between FA and postoperative changes in motor symptoms were identified. This relationship between regional mean fractional anisotropy and motor symptoms is detailed in Supplementary Table 1 and Supplementary Fig. 1.

### Interaction between NODDI-parameters and postoperative change in motor symptom burden

**Fig. 2:**
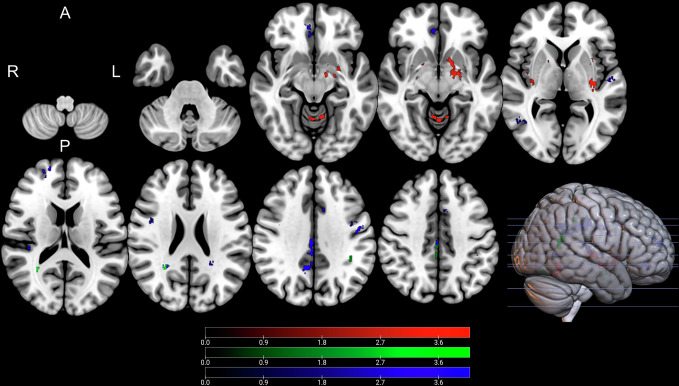
Clusters with a positive association between PD patients’ ODI-values and postoperative change in UPDRS (red), time spent in ON (green), and time spent in OFF (blue), as revealed by the whole brain analysis. Clusterwise P-values were corrected for multiple comparisons using a permutation-based approach and shown as the negative decadic logarithm of the p-value.

**Fig. 3:**
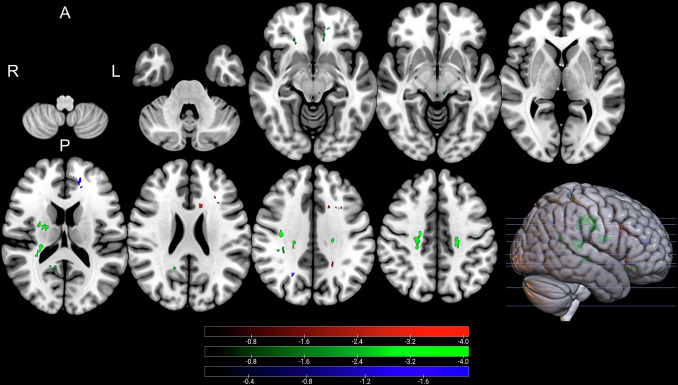
Clusters with a negative association between PD patients’ ODI-values and postoperative change in UPDRS (red), time spent in ON (green), and time spent in OFF (blue), as revealed by the whole brain analysis. P-values were corrected for multiple comparisons using a permutation-based approach. Clusterwise P-Values were corrected for multiple comparisons using a permutation-based approach and shown as the negative decadic logarithm of the p-value.

Whole brain analysis of NODDI parameters indicated that both ODI and NDI were significantly correlated with changes in postoperative UPDRS part III scores. These findings are illustrated in Supplementary Tables 2 and 3 as well as Figs. 2 and 3 (ODI) and Supplementary Figs. 3 and 4 (NDI values). Higher ODI values in the left pallidum, bilateral putamen, and bilateral insular cortex were associated with greater change in postoperative motor symptoms (P1-6, CWP: <0.001–0.038, Fig. 2). Conversely, negative associations were exclusively observed in white matter regions adjacent to the left middle frontal gyrus, left cingulate gyrus, and left precuneus (N1-3, CWP: <0.001-0.019, Fig. 3). Furthermore, higher ODI and NDI values in the bilateral insular cortex were related to a greater reduction in postoperative motor symptoms (ODI: P3, P5, CWP: <0.001-.003; NDI: P1–P3, CWP: <0.001–0.032). Additionally, increased NDI values in the left putamen were liked to greater reduction in postoperative motor symptoms (P1, CWP: <0.001). Negative associations between NDI and postoperative motor symptoms were identified in white matter regions adjacent to the left middle frontal gyrus, left precentral gyrus, right precuneus, and left paracingulate gyrus, corresponding to the previously mentioned negative ODI-clusters (N1-3, CWP: <0.001−0.019).

### Interaction between microstructural metrics and time with good/poor mobility

Regional mean values of microstructural metrics associated with significant postoperative variations in time spent in the ON and OFF states are presented in Supplementary Tables 4 and 5, as well as Supplementary Figs. 3 and 4. Decreased values of microstructural metrics within gray matter regions of the right pre- and postcentral gyrus and the right precuneus were correlated with increased time spent in the ON state (ODI: N3, N6, N7, CWP: <0.001–0.002). No corresponding negative NDI clusters were identified. Regarding the time spent in the OFF state, associations across extensive gray matter regions were observed. Specifically, higher ODI values in the right cingulate gyrus, left pre- and postcentral gyrus, and the right precuneus were related to greater postoperative changes in time spent in OFF state (ODI: P1-3, CWP: <0.001). Additionally, elevated NDI values in the left middle frontal gyrus and the left precentral gyrus were associated with greater postoperative changes in time spent in the OFF state (NDI: P1, P4, CWP: <0.001–0.01).

### Interaction between microstructure and postoperative motor outcomes controlled for medication, age, and disease duration

In a supplementary analysis, we reran the GLM analysis and assessed the relationship between DTI metrics and postoperative motor outcomes (UPDRS-III, time spent in good mobility without troublesome dyskinesia, time spent in poor mobility) while considering LEDD, age, and disease duration as a covariate. For conciseness, findings are displayed in Supplementary Figs. 6–11. While certain smaller clusters and clusters within left pre- and postcentral gyrus associated with poor mobility did not survive correction for multiple comparisons when accounting for the covariates named above, we could replicate the main findings of our analysis for UPDRS part III as well as the time spent in good and poor mobility.

## Discussion

In the present study, we employed NODDI-DTI, an innovative method for analyzing diffusion-weighted imaging (DWI) data, to investigate the association between cerebral microstructure and alterations in motor symptoms following subthalamic stimulation in patients with PD. Our findings can be summarized in three key points. First, we demonstrate that intact microstructure within the bilateral putamen, bilateral insula, and left pallidum is associated with a greater reduction in postoperative motor symptoms. Second, we identify specific white matter tracts whose selective degeneration correlates with below-average improvements in postoperative motor symptoms. Third, we find that preserved microstructure in certain brain structures is linked to postoperative increases in the duration of time spent in good mobility and no troublesome dyskinesia, as well as decreases in the time spent in poor mobility.

Parkinson’s disease is characterized by the cardinal symptoms of bradykinesia, rigidity, and tremor, which significantly impair the quality of life of affected individuals^16,17^. These debilitating symptoms are evaluated using the UPDRS to gauge the overall motor burden^18^. The UPDRS part III has been established as the standard parameter for overall motor symptom severity in PD and serves as a primary outcome measure in numerous clinical trials^19–21^. In the present study, whole-brain analysis of NODDI parameters identified an association between higher ODI values within bilateral putamen, bilateral insula, and left pallidum and a higher reduction in postoperative UPDRS part III values. Given that ODI is high in gray matter^12^, this finding supports the hypothesis that the integrity of dendritic processes in these regions is pivotal for achieving beneficial postoperative motor outcomes.

Given the intricate relationship between the underlying disease mechanisms and the physiological effects of the intervention, several aspects have to be considered when interpreting the present findings. First, the pathological accumulation of alpha-synuclein in intraneuronal Lewy inclusions results in neuronal degeneration and significant morphological changes in dendrites^22,23^. These tissue alterations are not detectable using conventional MRI, whereas NODDI is sensitive to variations in neurite morphology. Previous studies have documented reduced putaminal ODI values in people with PD compared to healthy controls, interpreted as indicative of decreased dendritic length and loss of spines in striatal medium spiny neurons, the primary targets of dopaminergic nigrostriatal projections. Therefore, it can be hypothesized that low ODI values in these structures reflect the compromised neurite morphology described by Kamagata as well as Schröder and colleagues^23,24^.

Second, given the close topographical relationship between the STN, the putamen, and the pallidum along with the integration of STN in basal ganglia-thalamo-cortical loops, it is essential to examine the putative mechanisms of action of DBS. Besides its effects at the micro- and mesoscale, the high-frequency electrical pulses from DBS electrodes influence interregional networks on a macroscale^25^. This network modulation has been shown to predict postoperative outcomes across both motor and various non-motor symptoms^26^. Therefore, it can be postulated that the positive association between ODI and postoperative motor outcomes in the present study reflects the reliance of DBS on intact tissue structure to exert its network effects. In this context, Hermann et al. emphasize the significance of preserved microstructure in motor regions such as the STN, substantia nigra (SN), and putamen for the efficacy of STN-DBS^27^. Using free interstitial fluid (V-CSF) as a marker of microstructure, the authors identified a correlation between V-CSF-values in their region of interest (ROI) and postoperative improvements in UPDRS part III. Despite notable effect sizes, these results did not reach statistical significance after adjusting for multiple comparisons. In the present study, we address the limitations of region of interest-based and correlation analyses by integrating a whole-brain approach with generalized linear modeling, thus partially confirming the association between intact putaminal microstructure and improvements in motor symptoms. Furthermore, a growing body of literature on structural imaging has identified other anatomical features as predictors of motor outcomes in Parkinson’s disease, including the mesencephalon surface, thalamic volumes, cortical thickness in the left lateral-occipital cortex and morphometry of the superior frontal cortex^28–31^. Collectively, these results underline the potential of structural imaging, and microstructural imaging in particular, to evaluate postoperative outcomes of neurostimulation in Parkinson’s disease.

Besides associations with areas of the basal ganglia-thalamo-cortical loop, ODI showed a positive association with post-operative motor burden in the bilateral insular cortex. The insular cortex has been primarily considered a visceral-somatic region within the limbic system^32^, and is a major target area of thalamocortical projections^33,34^. Studies suggest a role for the insular cortex in integrating autonomic, cognitive-affective, and somatosensory information, mediating the control of non-motor symptoms in PD^35,36^. The authors point to the pathological accumulation of alpha-synuclein^37^ as a cause of altered insular function, subsequently leading to non-motor symptoms such as aberrant sensory processing and urinary issues. Conversely, recent studies have demonstrated an influence of the insular cortex on gating mechanisms in motor control. Wu and colleagues found decreased functional connectivity from the pre-supplementary motor area to the mid-anterior insula in patients with PD^38^. These disrupted connections indicate a lack of readiness for movement and may partly contribute to the difficulty in initiating movements in PD. Extending this hypothesis, Tinaz et al. proposed a circuit model highlighting the interaction between the insula and the dorsomedial frontal cortex (harboring supplementary motor area (SMA) and pre-SMA) for generating intentional movements^39^. In this model, the insula processes afferent viscerosensory and somatosensory information from the body and integrates them with motivational and emotional context. This information is conveyed to the frontal cortex, which is involved in various higher-level cognitive functions^40–42^, and is supposed to generate the impetus to move. The authors conclude that altered insular function in PD patients contributes to the difficulty in internally generating intentional movements and in maintaining the speed, size, and vigor of movements^39^. Regarding the effects of STN-DBS on the insular cortex, Herzog et al. investigated the sensory gating of urinary bladder afferents following STN-DBS^43^. They demonstrated modulation of activity in the insular cortex and thalamus by STN-DBS, suggesting that partially restoring basal ganglia function via DBS is beneficial for sensory gating and urinary outcomes in PD. Integrating our findings with these studies, we suggest that the positive association between ODI and postoperative motor outcomes reflects the dependency of DBS on intact tissue structure within the bilateral insula to exert beneficial effects on gating processes essential for movement control.

Whole brain analysis of NODDI parameters identified an association between lower FA- and NDI-values in several WM tracts, including the cingulum and a detrimental motor response to STN-DBS. In healthy white matter, NDI is usually high, as fibers are coherent; low NDI values represent axonal loss and degeneration^12^. Conversely, ODI in white matter is typically low. As most negative FA- and NDI-clusters overlapped with clusters where ODI showed a negative association with postoperative motor symptom burden and lay within regions of high fiber crossing and dispersion, the selective degeneration of crossing fibers might underlie the observed relationship. The results, therefore, support the hypothesis that intact microstructure of the cingulum and its crossing fibers is important for beneficial postoperative changes in motor symptoms.

This study employed whole-brain analysis of NODDI parameters to identify significant associations between favorable motor states and the integrity of microstructural features within the bilateral pre- and postcentral gyri, right precuneus, and cingulum. In particular, an increased duration of time spent in the ON-state associated with preserved microstructural integrity, as indicated by the ODI, in the right pre- and postcentral gyri and the right precuneus. Furthermore, a decrease in the duration of time spent in the OFF-state following STN-DBS was associated with intact microstructure in the right cingulate gyrus, left pre- and postcentral gyri, and right precuneus. Numerous functional MRI (fMRI) studies have shown that neurostimulation significantly impacts the connectivity of the primary motor cortex (for a review see Miao et al. and Li et al.)^44,45^. These studies provide evidence that STN-DBS alters the resting-state functional connectome, enhancing coupling within the direct pathway while reducing coupling in the hyperdirect pathway. Furthermore, the connectivity between DBS electrodes and a network of brain regions, including the primary motor cortex, is predictive of clinical responses to STN-DBS^46^. Integrating these studies with the fact that the pre- and postcentral gyrus contain structures essential for voluntary motor control and somatosensory information processing, it is conceivable that intact microstructure within these areas is essential for the efficacy of DBS.

It is important to acknowledge that this research is not without its limitations. First, while the NODDI model is histopathologically validated and widely used in PD research, there are currently no studies validating it specifically in the context of PD. Second, the fundamental principles of the NODDI model may oversimplify the situation, potentially reducing specificity. Third, the resolution of the DTI scan in our study is limited to 2.0 × 2.0 × 2.0 mm³ and 42 diffusion gradients. This resolution and number of gradients, however, is similar to previous NODDI studies^47–49^ and was chosen to find a compromise between scanning time, image resolution, and signal-to-noise ratio, where longer durations in the scanner lead to more noticeable motion artefacts, a crucial consideration in PD. Fourth, the general limitations of open-label studies apply here. While open-label designs have advantages, they also present limitations, including potential placebo effects that may influence subjective measures and patient well-being. Furthermore, subjective measures such as patient diaries can be prone to inaccuracies, as patients may be unable to correctly recognize or report their symptoms. In this context, it is important to note that in the present study, the UPDRS part II, which assesses patients’ motor experiences of daily living, showed no significant difference compared to baseline during the best condition. This result aligns with findings from a large randomized controlled trial, which also reported no significant changes in UPDRS part II scores when assessed under the best condition^50^. Finally, a control group cannot be included in open-label DBS studies. To mitigate these challenges and reduce bias, several measures were implemented. First, the primary outcomes were selected to include a combination of clinician-rated and patient-reported subjective outcomes. Second, patient-reported outcomes were assessed using standardized instructions and case report forms. Third, the UPDRS part III was evaluated by a single rater blinded to whether the videos were from baseline or follow-up visits, using standardized video recordings. Fourth, assessments from 3- and 12-month follow-up visits demonstrated consistent effects, similar to those observed at the six-month follow-up. Fifth, a complementary analysis accounting for potential confounding variables, including LEDD, age, and disease duration, confirmed the robustness of our findings.

In conclusion, we identified a distinct spatial profile of microstructural alterations associated with motor outcomes following neurostimulation in PD. In particular, intact microstructure within the bilateral putamen, bilateral insula, and left pallidum were associated with a higher reduction in postoperative motor symptoms. Furthermore, preserved microstructure in pre- and postcentral gyrus and right precuneus and cingulum was associated with increases in time spent in ON as well as decreases in time spent in OFF. These findings remain consistent even after accounting for LEDD, age, and disease duration. Our results highlight the possibility of preoperatively assessing microstructural alterations to support patient counseling and treatment planning by identifying patients likely to experience above- or below-average motor responses.

## Methods

### Participants

In this ongoing observational study, thirty-seven PD patients were enrolled upon written informed consent. Inclusion criteria comprised indication for DBS surgery due to advanced PD following international criteria, including the presence of motor fluctuations and/or dyskinesias^51^. Patients with pathological MRI, concurrent neurological or psychiatric conditions, or impaired visual or auditory function were excluded. Two patients were excluded due to missing follow-up data. The present study includes a cohort of participants that partially overlaps with those reported in our previous publication, as part of the ongoing observational study. The study was conducted according to the Declaration of Helsinki and approved by the Philipps-University of Marburg ethics committee (study-number: 155/17).

### Clinical assessment

Patients were evaluated at the preoperative baseline and at six months after DBS lead surgery. Both study visits were conducted while the patients were in the ON-medication state, whereas follow-up assessments were performed in the ON-medication/ON-stimulation state (MedON/StimON). Neurosurgical standards were defined by the involved neurosurgeons to guarantee an optimal approach and were consistent with the methodology described by Deuschl et al.^52^. Standardized case report forms were utilized to gather demographic and clinical data, including the UPDRS, the PDQ-8, and overall mobility. The Unified Parkinson’s Disease Rating Scale part III (UPDRS-III) is a clinician-rated scale evaluating motor symptom severity ranging from 0 (no impairment) to 108 (maximum impairment)^18^. The UPDRS-III was evaluated by a single rater (PAL) based on standardized video recordings, except for the rigidity items, which cannot be evaluated via video analysis. The rater was blinded to whether the videos originated from baseline or follow-up visits. The PDQ-8 is a validated tool for assessing quality of life across eight dimensions in PD patients undergoing DBS surgery. It provides a summary index (SI) score ranging from 0 (no impairment) to 100 (maximum impairment)^53^. Mobility data were recorded in a patient diary, documenting the number of hours per day spent in good mobility and without troublesome dyskinesia (ON), poor mobility (OFF), good mobility with troublesome dyskinesia (ON TD), and sleep (SLEEP)^54^. Patients were instructed to complete these diaries for the 36 h preceding the study visit. The LEDD was calculated according to the methodology described by Jost et al.^55^. To ensure a consistent postoperative adjustment of medication and mitigate a potential confounding effect of LEDD on motor outcomes, we implemented a standardized protocol for reducing LEDD after DBS implantation, aligning with the approach used in the EARLYSTIM study^52^. Likewise, the adjustment of stimulation parameters was performed using a standardized protocol, also based on the methodology employed in the EARLYSTIM study^52^. Demographic and clinical characteristics are summarized in Table 1 and Fig. 1.

To increase the reliability of our clinical data and minimize the potential influence of symptom fluctuations caused by varying stressors, we performed a supplementary analysis incorporating data from the three- and twelve-month follow-up visits.

### MRI data acquisition and processing

At preoperative baseline, PD patients were scanned at the University of Marburg’s Core Unit Brain Imaging using a 3-Tesla Trio scanner (Siemens, Erlangen, Germany). During image acquisition, patients were awake and in the ON-medication state. The acquisition protocol comprised the following sequences:

1. 3D T1-weighted Magnetization Prepared - RApid Gradient Echo sequence (MPRAGE, field of view (FoV) = 256 mm, matrix 256 × 256, 176 slices, slice thickness 1 mm, voxel dimension 1.0 × 1.0 × 1.0 mm³, repetition time (TR) = 1900 ms, echo time (TE) = 2.26 ms, inversion time (TI) = 900 ms, flip-angle = 9°, bandwidth (BW) = 200 Hz/Pixel, parallel imaging (GRAPPA) with factor 2).
2. diffusion weighted imaging (DWI) (FoV = 256 mm, matrix 128 × 128, slice thickness 2 mm, distance factor 0%, voxel dimension 2.0 × 2.0 × 2.0 mm³, TR = 7900 ms, TE = 90 ms, BW = 1502 Hz/Pixel, 42 diffusion encoding gradients, three intermittent non-weighted b0 images (*b* = 0 s/mm²), high b-value b = 1000 s/mm², GRAPPA with factor 2).

All images were investigated to be free of motion or ghosting and high frequency and/or wrap-around artefacts at the time of image acquisition. Postoperative evaluation included a computed tomography (CT) scan to assess for complications such as intracranial bleeding, edema, or lead misplacement. No postoperative complications were identified on imaging.

### Image Processing

Analysis of structural images was performed within the FreeSurfer image analysis suite version 7.1.1 (http://surfer.nmr.mgh.harvard.edu), as previously reported by our research group^15^. The processing pipeline comprised several steps including skull stripping, automated Talairach transformation, cortical and subcortical segmentation, intensity normalization, tessellation of the gray/white matter boundary, automated topology correction, and surface deformation based on intensity gradients.

The analysis of diffusion-weighted images was carried out within the FMRIB Software Library (FSL) analysis suite version 6.0.5.2 (https://fsl.fmrib.ox.ac.uk/fsl). Raw DWI volumes were registered and resampled to the first b0 volume to correct for eddy-current distortions and involuntary movements. Subsequently, the diffusion tensor for each voxel was estimated using linear regression from which FA was derived. Additionally, NODDI-DTI, a modification of NODDI, was employed to obtain NDI and ODI from the DTI data. Visual inspection of the b0 images confirmed that no alterations beyond those inherent in the tissue structure contributed to the observed effects. Regional analyses were performed by linearly registering the first b0 image of each scan to the structural T1-weighted image using a boundary-based registration method and resulting in an affine matrix. By applying the inverse of this matrix, the T1-derived segmentations and brain masks were transformed into diffusion space. With the aid of both linear and nonlinear transformations, FA-maps were initially co-registered to the MNI152 standard space^56^. Subsequently, masked FA-, NDI-, and ODI-maps were registered to the MNI152 space using the transformation established in the preceding step, excluding voxels of brain tissue that were not consistently present across all subjects from the analysis.

To verify the correct placement of the electrodes, we performed a supplementary analysis using the Lead-DBS toolbox with default parameters (www.lead-dbs.org). Briefly, advanced normalization tools (ANTs, http://stnava.github.io/ANTs/) were employed to linearly coregister postoperative CT images with preoperative MRI. The images were then nonlinearly normalized to standard space (ICBM 2009b NLIN, Asym), and the PaCER algorithm was utilized for electrode reconstruction (Supplementary Fig. 5). This approach allowed us to confirm accurate lead placement in the participants.

### Statistical analysis

Statistical analysis of clinical outcomes was conducted utilizing MATLAB R2020b (The MathWorks, Inc.). Mean changes in clinical scores between baseline and the 6-month follow-up (6-MFU) were evaluated using Wilcoxon signed-rank-test or t-tests, according to results of the Shapiro-Wilk test for normality assessment. The false discovery rate was controlled by employing the Benjamini-Hochberg method, and effect sizes were calculated in accordance with Cohen’s conventions^57,58^. Reported p-values are two-sided and were deemed significant at *p* < 0.05.

Statistical voxelwise analysis of imaging data was performed using the tool mri_glmfit of FreeSurfer. In this analysis, clinical outcomes were represented as percentage difference in UPDRS-III scores, time spent in good mobility without troublesome dyskinesia, and time spent in poor mobility. Associations between microstructure and time spent in good mobility with troublesome dyskinesia (ON TD) were not performed due to the small number of patients experiencing dyskinesia (*n* = 14).

Associations between metrics of microstructure and changes in clinical outcomes were conducted across the whole brain using a generalized linear model^59^. A permutation-based approach utilizing the Analysis of Functional NeuroImages (AFNI) null-z simulator, was employed to correct for multiple comparisons using 12,000 simulations under the null hypothesis^60^. Clusters were formed using a threshold of *p* < 0.01, and a clusterwise p-value was calculated. Results were accepted as significant when the clusterwise *p* < 0.05. In a complementary analysis, we reran the statistical voxelwise analysis incorporating LEDD, age, and disease duration as a covariate (c.f. Supplementary Material).

## Acknowledgement

The authors would like to thank the participants for their active engagement in this study.

## Supplementary information

Supplementary information

## Data Availability

The data that support the findings of this study is available on request from the corresponding author (PAL). The data is not publicly available due to privacy or ethical restrictions. All tools used for the analysis of MRI data are based on FreeSurfer Version 7.1 (http://surfer.nmr.mgh.harvard.edu/) and FSL 6.0.5.2 (http://www.fmrib.ox.ac.uk/fsl) packages, which are freely available. Scripts for automation were written in tcshell and parts of the statistics were written in Python using the packages numpy, pandas, seaborn, matplotlib, nibabel and scipy, which are also freely available. Python program code for the analysis of NODDI-DTI is available from https://github.com/dicemt/DTI-NODDI.

## Abbreviations

6-MFU: 6-month follow-up
LEDD: Levodopa equivalent daily dose
LEDD-DA: LEDD of Dopamine Agonists
M: mean
OFF: time spent in poor mobility
ON: time spent in good mobility and without troublesome dyskinesia
ON TD: time spent in good mobility with troublesome dyskinesia
PDQ-8 SI: 8-item Parkinson’s Disease Questionnaire summary index
SD: standard deviation
SLEEP: time allocated to sleep
UPDRS: Unified Parkinson’s Disease Rating Scale

## Code availability

The data that support the findings of this study is available on request from the corresponding author (PAL). The data is not publicly available due to privacy or ethical restrictions. All tools used for the analysis of MRI data are based on FreeSurfer Version 7.1 (http://surfer.nmr.mgh.harvard.edu/) and FSL 6.0.5.2 (http://www.fmrib.ox.ac.uk/fsl) packages, which are freely available. Scripts for automation were written in tcshell and parts of the statistics were written in Python using the packages numpy, pandas, seaborn, matplotlib, nibabel and scipy, which are also freely available. Python program code for the analysis of NODDI-DTI is available from https://github.com/dicemt/DTI-NODDI.
