# Supplementary material for: Microstructure is associated with motor outcomes following Deep Brain Stimulation in Parkinson’s disease
Source: NPJ Parkinsons Dis. 2025 Apr 21;11:81. doi: 10.1038/s41531-025-00930-3 (PMC12012084; doi:10.1038/s41531-025-00930-3)
Supplement: Supplementary file 1 — Supplementary information [file 41531_2025_930_MOESM1_ESM.docx]

# Supplementary Material

**Supplementary Results**

**Image analysis**

**Supplementary Table 1 Association between FA-Values and UPDRS part III**

| Negative Cluster | Location | Slope | Intercept | p-Value | Volume in mm³ | MNI152-Coordinates | | |
| --- | --- | --- | --- | --- | --- | --- | --- | --- |
|  |  |  |  |  |  | X | Y | Z |
| N1 | Right inferior fronto-occipital fasciculus  Right putamen | -.0005 | .28503 | < .001 | 341 | 28 | -5 | -8 |
| N2 | Left superior longitudinal fasciculus | -.0005 | .51221 | < .001 | 330 | -39 | -39 | 14 |
| N3 | Left V | -.0003 | .12832 | .009 | 208 | -19 | -48 | -11 |
| N4 | Left superior frontal gyrus | -.0005 | .48711 | .027 | 186 | -19 | 31 | 25 |
| N5 | Right inferior Frontal Gyrus | -.0004 | .39693 | .032 | 183 | 33 | 15 | 16 |
| N6 | Right forceps minor | -.0006 | .23986 | .033 | 182 | 13 | 40 | -10 |
| N7 | Right cingulate gyrus, anterior division | -.0005 | .27215 | .035 | 181 | 14 | 17 | 28 |

**Supplementary Table 1.** Characteristics of clusters with an association between PD patients’ FA-values and postoperative change in UPDRS part III. “Negative Cluster” denotes clusters with a negative association between patients’ FA-values and percentage difference of UPDRS part III, i.e. higher FA-values were associated with lower postoperative values. “Location” indicates the anatomical landmark comprising the majority of voxels of a cluster according to Johns Hopkins University (JHU) white matter atlas, Harvard-Oxford cortical and subcortical atlas, and University College London (UCL) cerebellar atlas. P-Values are clusterwise p-values corrected for multiple comparisons. “Volume in mm³” denotes the size of a cluster and “MNI152-coordinates” describes the coordinates of the cluster’s center of gravity in MNI152-space.

**Supplementary Table 2 Association between ODI-Values and UPDRS part III**

| Positive  Cluster | Location | Slope | Intercept | p-Value | Volume in mm³ | MNI152-Coordinates | | |
| --- | --- | --- | --- | --- | --- | --- | --- | --- |
|  |  |  |  |  |  | X | Y | Z |
| P1 | Bilateral V | .0008 | .2511 | < .001 | 393 | -1 | -59 | -5 |
| P2 | Left pallidum Left putamen | .0004 | .1701 | < .001 | 360 | -22 | -8 | -8 |
| P3 | Left insular cortex | .0005 | .1938 | < .001 | 298 | -34 | -18 | 1 |
| P4 | Left insular cortex Left putamen Left inferior fronto-occipital fasciculus | .0004 | .2424 | .003 | 173 | -30 | 7 | 3 |
| P5 | Right insular cortex | .0005 | .1883 | .003 | 172 | 33 | -17 | 3 |
| P6 | Right putamen | .0006 | .2546 | .038 | 135 | 17 | 3 | -5 |
| Negative Cluster |  |  |  |  |  |  |  |  |
|  |  |  |  |  |  |  |  |  |
| N1 | Left cerebral white matter adjacent to left middle frontal gyrus | -.0005 | .2852 | < .001 | 222 | -32 | 19 | 23 |
| N2 | Left cerebral white matter adjacent to left cingulate gyrus | -.0003 | .1587 | .001 | 195 | -17 | 16 | 26 |
| N3 | Left cerebral white matter adjacent to left precuneal gyrus | -.0004 | .1967 | .019 | 145 | -21 | -48 | 35 |

**Supplementary Table 2.** Characteristics of clusters with an association between PD patients’ ODI-values and postoperative change in UPDRS part III. “Positive Cluster” denotes clusters with a positive association between patients’ ODI-values and percentage differences in UPDRS part III, i.e. higher ODI-values were associated with higher postoperative values. “Negative Cluster” denotes clusters with a negative association between patients’ ODI-values and percentage difference of UPDRS part III, i.e. higher ODI-values were associated with lower postoperative values. “Location” indicates the anatomical landmark comprising the majority of voxels of a cluster according to Johns Hopkins University (JHU) white matter atlas, Harvard-Oxford cortical and subcortical atlas, and University College London (UCL) cerebellar atlas. P-Values are clusterwise p-values corrected for multiple comparisons. “Volume in mm³” denotes the size of a cluster and “MNI152-coordinates” describes the coordinates of the cluster’s center of gravity in MNI152-space.

**Supplementary Table 3 Association between NDI-Values and UPDRS part III**

| Positive  Cluster | Location | Slope | Intercept | p-Value | Volume in mm³ | MNI152-Coordinates | | |
| --- | --- | --- | --- | --- | --- | --- | --- | --- |
|  |  |  |  |  |  | X | Y | Z |
| P1 | Left insular cortex Left Putamen Left inferior fronto-occipital fasciculus | .0008 | .4811 | < .001 | 237 | -35 | -18 | 0 |
| P2 | Left insular cortex | .0008 | .3738 | .007 | 158 | -32 | 19 | -2 |
| P3 | Right insular cortex | .0009 | .2633 | .032 | 131 | 38 | 7 | -5 |
| P4 | Left cerebral white matter adjacent to anterior commissure | .0008 | .4519 | .037 | 129 | -13 | -10 | -10 |
| Negative Cluster |  |  |  |  |  |  |  |  |
|  |  |  |  |  |  |  |  |  |
| N1 | Left superior longitudinal fasciculus | -.0005 | .5344 | < .001 | 778 | -40 | -46 | 17 |
| N2 | Left cerebral white matter adjacent to superior parietal lobule  Left anterior thalamic radiation | -.0006 | .4971 | < .001 | 505 | -22 | -49 | 34 |
| N3 | Left superior longitudinal fasciculus Left middle temporal gyrus | -.0005 | .5662 | < .001 | 299 | -45 | -47 | 2 |
| N4 | Left superior longitudinal fasciculus adjacent to precentral gyrus | -.0006 | .6200 | < .001 | 273 | -39 | -3 | 24 |
| N5 | Left cerebral white matter adjacent to left middle frontal gyrus | -.0007 | .6071 | < .001 | 250 | -30 | 15 | 23 |
| N6 | Left corticospinal tract Left precentral gyrus | -.0008 | .5884 | < .001 | 243 | -23 | -9 | 41 |
| N7 | Right forceps major Right inferior fronto-occipital fasciculus | -.0005 | .4564 | .002 | 178 | 30 | -51 | 17 |
| N8 | Right inferior fronto-occipital fasciculus adjacent to right precuneal cortex | -.0005 | .5205 | .004 | 166 | 15 | -62 | 40 |
| N9 | Left cerebral white matter adjacent to left paracingulate gyrus Left paracingulate gyrus | -.0010 | .5903 | .006 | 159 | -13 | 29 | 28 |
| N10 | Right inferior longitudinal fasciculus adjacent to lateral occipital cortex | -.0005 | .5621 | .015 | 144 | 32 | -77 | 0 |
| N11 | Left cerebral white matter adjacent to inferior frontal gyrus inferior frontal gyrus | -.0007 | .5747 | .023 | 137 | -40 | 24 | 14 |

**Supplementary Table 3.** Characteristics of clusters with an association between PD patients’ NDI-values and postoperative change in UPDRS part III. “Positive Cluster” denotes clusters with a positive association between patients’ NDI-values and percentage differences in UPDRS part III, i.e. higher NDI-values were associated with higher postoperative values. “Negative Cluster” denotes clusters with a negative association between patients’ NDI-values and percentage difference of UPDRS part III, i.e. higher NDI-values were associated with lower postoperative values. “Location” indicates the anatomical landmark comprising the majority of voxels of a cluster according to Johns Hopkins University (JHU) white matter atlas, Harvard-Oxford cortical and subcortical atlas, and University College London (UCL) cerebellar atlas. P-Values are clusterwise p-values corrected for multiple comparisons. “Volume in mm³” denotes the size of a cluster and “MNI152-coordinates” describes the coordinates of the cluster’s center of gravity in MNI152-space.

**Mobility specific Statistics**

**Supplementary Table 4 Association between microstructural metrics and time with good mobility and no dyskinesia**

| **Fractional Anisotropy** | | |  |  |  |  | | |
| --- | --- | --- | --- | --- | --- | --- | --- | --- |
| Positive  Cluster | Location | Slope | Intercept | p-Value | Volume in mm³ | MNI152-Coordinates | | |
|  |  |  |  |  |  | X | Y | Z |
| P1 | Left superior longitudinal fasciculus Left postcentral gyrus Left corticospinal tract | .0005 | .4836 | < .001 | 3311 | -34 | -13 | 29 |
| P2 | Right corticospinal tract | .0004 | .4397 | < .001 | 684 | 21 | -25 | 35 |
| P3 | Right inferior fronto-occipital fasciculus | .0003 | .4198 | < .001 | 509 | 26 | 25 | 8 |
| P4 | Right insular cortex  Right inferior fronto-occipital fasciculus | .0004 | .3846 | < .001 | 474 | 36 | -10 | -1 |
| P5 | Right frontal orbital cortex | .0005 | .3055 | < .001 | 467 | 24 | 9 | -19 |
| P6 | Right forceps minor | .0005 | .4731 | < .001 | 395 | 19 | 35 | 1 |
| P7 | Right superior longitudinal fasciculus Right precentral gyrus Right postcentral gyrus | .0005 | .4777 | < .001 | 330 | 40 | -13 | 21 |
| P8 | Right superior longitudinal fasciculus Right postcentral gyrus | .0005 | .5358 | < .001 | 284 | 33 | -30 | 37 |
| P9 | Left superior longitudinal fasciculus | .0005 | .5317 | < .001 | 273 | -27 | -8 | 16 |
| P10 | Right forceps minor Right frontal pole | .0005 | .4543 | < .001 | 259 | 19 | 41 | -8 |
| P11 | Left cingulate gyrus, posterior division | .0005 | .4931 | .002 | 252 | -8 | -33 | 30 |
| P12 | Left forceps minor Left frontal pole | .0006 | .3119 | .002 | 249 | -14 | 51 | 0 |
| P13 | Right forceps major Right inferior fronto-occipital fasciculus | .0006 | .4953 | .002 | 241 | 31 | -58 | 20 |
| P14 | Left superior longitudinal fasciculus Left paracingulate gyrus | .0005 | .2271 | .004 | 232 | -12 | 11 | 39 |
| P15 | Left anterior thalamic radiation Left thalamus | .0004 | .3514 | .005 | 230 | -12 | -25 | -7 |
| P16 | Left superior longitudinal fasciculus Left superior temporal gyrus | .0004 | .4771 | .007 | 222 | -32 | -39 | 20 |
| P17 | Right superior longitudinal fasciculus Right superior parietal lobule | .0003 | .4381 | .013 | 206 | 22 | -40 | 40 |
| P18 | Right cerebral white matter adjacent to cingulate gyrus, anterior division | .0004 | .4880 | .013 | 204 | 15 | 7 | 33 |
| P19 | Left inferior fronto-occipital fasciculus Left insular cortex | .0003 | .4286 | .031 | 185 | -24 | 24 | 9 |
| P20 | Right corticospinal tract | .0004 | .4921 | .045 | 176 | 27 | -23 | 23 |
| P21 | Right superior frontal gyrus | .0009 | .4086 | .046 | 175 | 12 | 10 | 50 |
| P22 | Left thalamus Left anterior thalamic radiation | .0004 | .3428 | .050 | 173 | -14 | -12 | 13 |
| **Orientation Dispersion Index** | | |  |  |  |  |  |  |
| Positive Cluster |  |  |  |  |  |  |  |  |
| P1 | Right inferior fronto-occipital fasciculus adjacent to precuneal cortex | .0002 | .1021 | < .001 | 273 | 29 | -51 | 17 |
| P2 | Right cingulate gyrus, posterior division | .0010 | .3157 | .009 | 158 | 0 | -24 | 39 |
| P3 | Right inferior fronto-occipital fasciculus adjacent to planum polare | .0005 | .2428 | .017 | 146 | -37 | -9 | -21 |
| P4 | Right cingulate gyrus, posterior division | .0009 | .3354 | .024 | 141 | 1 | -34 | 39 |
| P5 | Left superior longitudinal fasciculus adjacent to supramarginal gyrus, anterior division | .0005 | .2765 | .024 | 141 | -41 | -37 | 32 |
| P6 | Left VI | .0007 | .3292 | .03 | 138 | -10 | -74 | -12 |
| Negative Cluster |  |  |  |  |  |  |  |  |
| N1 | Right corticospinal tract | -.0003 | .1600 | < .001 | 495 | 20 | -23 | 35 |
| N2 | Right superior longitudinal fasciculus | -.0003 | .1779 | < .001 | 396 | 28 | -2 | 18 |
| N3 | Right superior longitudinal fasciculus adjacent to right pre- and postcentral gyrus | -.0004 | .1385 | < .001 | 262 | 34 | -15 | 33 |
| N4 | Right inferior fronto-occipital fasciculus | -.0003 | .1145 | < .001 | 258 | 26 | -30 | 13 |
| N5 | Left corticospinal tract | -.0003 | .1438 | < .001 | 235 | -23 | -20 | 34 |
| N6 | Right cerebral white matter adjacent to right precuneal cortex and right cingulum | -.0004 | .1903 | .002 | 190 | 14 | -48 | 20 |
| N7 | Right superior longitudinal fasciculus adjacent to right postcentral gyrus | -.0003 | .1195 | .002 | 188 | 36 | -31 | 29 |
| N8 | Left anterior thalamic radiation Left thalamus | -.0004 | .3072 | .004 | 175 | -11 | -27 | -1 |
| N9 | Left forceps minor  Left frotal medial cortex | -.0004 | .2098 | .018 | 146 | -12 | 40 | -14 |
| N10 | Right cerebral white matter adjacent to frontal orbital cortex | -.0004 | .2159 | .032 | 136 | 21 | 28 | -11 |
| **Neurite Density Index** | | |  |  |  |  |  |  |
| Positive Cluster |  |  |  |  |  |  |  |  |
| P1 | Left superior longitudinal fasciculus adjacent to left postcentral gyrus | .0005 | .5494 | < .001 | 568 | -46 | -16 | 25 |
| P2 | Left middle temporal gyrus Left superior longitudinal fasciculus adjacent to left middle temporal gyrus | .0007 | .5078 | < .001 | 364 | -52 | -60 | 0 |
| P3 | Right forceps major | .0005 | .3841 | < .001 | 301 | 32 | -51 | 11 |
| P4 | Left thalamus Left anterior thalamic radiation | .0006 | .5911 | < .001 | 244 | -11 | -10 | 9 |
| P5 | Left superior longitudinal fasciculus adjacent to left central opercular cortex and left post- and precentral cortex | .0006 | .5727 | < .001 | 239 | -42 | -8 | 22 |
| P6 | Middle temporal gyrus, posterior division | .0007 | .5299 | < .001 | 226 | -50 | -35 | -4 |
| P7 | Left precentral gyrus Left superior longitudinal fasciculus | .0009 | .5227 | < .001 | 205 | -42 | 2 | 22 |
| P8 | Left superior longitudinal fasciculus adjacent to left pre- and postcentral gyrus | .0005 | .6022 | .003 | 168 | -31 | -18 | 39 |
| P9 | Right frontal orbital cortex | .0012 | .4402 | .005 | 163 | 24 | 9 | -19 |
| P10 | Left cerebral white matter adjacent to left cingulate gyrus, posterior division | .0006 | .5436 | .011 | 149 | -14 | -19 | 28 |
| P11 | Left paracingulate gyrus | .0015 | .5512 | .012 | 147 | -10 | 11 | 39 |
| P12 | Left inferior longitudinal fasciculus adjacent to lateral occipital cortex, superior division | .0005 | .4860 | .032 | 131 | -31 | -63 | 23 |
| P13 | Left superior longitudinal fasciculus adjacent to precentral gyrus | .0005 | .6070 | .048 | 125 | -21 | -10 | 43 |

**Supplementary Table 4.** Characteristics of clusters with an association between PD patients’ microstructural metrics and postoperative change in time spent with good mobility and no dyskinesia (ON). “Positive Cluster” denotes clusters with a positive association between patients’ microstructural metrics and postoperative changes in time spent in ON, i.e. higher values of a specific metric were associated with higher postoperative values. “Negative Cluster” denotes clusters with a negative association between patients’ microstructural metrics and postoperative difference in time spent in ON, i.e. higher values of a specific metric were associated with lower postoperative values. “Location” indicates the anatomical landmark comprising the majority of voxels of a cluster according to Johns Hopkins University (JHU) white matter atlas, Harvard-Oxford cortical and subcortical atlas, and University College London (UCL) cerebellar atlas. P-Values are clusterwise p-values corrected for multiple comparisons. “Volume in mm³” denotes the size of a cluster and “MNI152-coordinates” describes the coordinates of the cluster’s center of gravity in MNI152-space.

**Supplementary Table 5 Association between microstructural metrics and time with poor mobility**

| **Fractional Anisotropy** | | |  |  |  |  | | |
| --- | --- | --- | --- | --- | --- | --- | --- | --- |
| Positive  Cluster | Location | Slope | Intercept | p-Value | Volume in mm³ | MNI152-Coordinates | | |
|  |  |  |  |  |  | X | Y | Z |
| P1 | Right forceps minor | .0002 | .3379 | < .001 | 262 | 19 | 36 | 8 |
| P2 | Left paracingulate gyrus | .0002 | .1517 | .002 | 241 | -10 | 9 | 43 |
| P3 | Right planum temporale | .0003 | .2119 | .005 | 222 | 50 | -33 | 16 |
| P4 | Left precentral gyrus and left superior longitudinal fasciculus adjacent to left pre- and postcentral gyrus | .0002 | .0751 | .005 | 221 | -49 | -8 | 31 |
| P5 | Right inferior fronto-occipital fasciculus | .0001 | .3929 | .048 | 172 | 26 | 25 | 10 |
| Negative Cluster |  |  |  |  |  |  |  |  |
|  |  |  |  |  |  |  |  |  |
| N1 | Left anterior thalamic radiation | -.0003 | .4682 | < .001 | 312 | -19 | -23 | 27 |
| N2 | Left corticospinal tract | -.0003 | .3922 | .001 | 251 | -22 | -9 | 26 |
| N3 | Corpus callosum | -.0002 | .8686 | .014 | 203 | -8 | -38 | 14 |
| N4 | Left temporal pole | -.0003 | .3042 | .033 | 183 | -34 | -3 | -24 |
| N5 | Right forceps major | -.0002 | .8510 | .039 | 178 | 16 | -45 | 13 |
| **Orientation Dispersion Index** | | |  |  |  |  |  |  |
| Positive Cluster |  |  |  |  |  |  |  |  |
| P1 | Right Cingulate gyrus, posterior division | .0004 | .2835 | < .001 | 377 | 3 | -21 | 34 |
| P2 | Left precentral gyrus Left postcentral gyrus Left superior longitudinal fasciculus adjacent to left pre- and postcentral gyrus | .0005 | .1738 | < .001 | 230 | -55 | -4 | 29 |
| P3 | Right precuneal cortex Right cingulate gyrus | .0005 | .2596 | < .001 | 213 | 8 | -55 | 31 |
| P4 | Right inferior fronto-occipital fasciculus | .0001 | .1017 | .001 | 198 | 24 | -48 | 28 |
| P5 | Right paracingulate gyrus | .0005 | .3507 | .001 | 195 | 5 | 38 | -8 |
| P6 | Right frontal pole Right forceps minor | .0004 | .2350 | .001 | 195 | 23 | 49 | 15 |
| P7 | Left anterior thalamic radiation | .0001 | .1204 | .001 | 189 | -25 | -46 | 21 |
| P8 | Right Heschl’s gyrus | .0003 | .2396 | .004 | 168 | 39 | -30 | 16 |
| P9 | Left paracingulate gyrus Left cingulate gyrus, anterior division | .0004 | .3059 | .008 | 159 | -8 | 11 | 39 |
| P10 | Left Heschl’s gyrus | .0003 | .2769 | .010 | 156 | -53 | -14 | 3 |
| P11 | Right middle temporal gyrus right superior longitudinal fasciculus | .0005 | .2830 | .023 | 142 | 47 | -60 | 3 |
| P12 | Right precentral gyrus Right superior longitudinal fasciculus | .0001 | .1894 | .023 | 142 | 41 | -1 | 23 |
| P13 | Left precentral gyrus Left superior longitudinal fasciculus | .0003 | .2062 | .039 | 133 | -46 | 4 | 37 |
| Negative Cluster |  |  |  |  |  |  |  |  |
| N1 | Left forceps minor adjacent to paracingulate gyrus | -.0002 | .2662 | .012 | 151 | -16 | 42 | 10 |
| N2 | Right inferior fronto-occipital fasciculus adjacent to precuneal cortex | -.0002 | .1804 | .023 | 141 | 20 | -59 | 37 |
| **Neurite Density index** | | |  |  |  |  |  |  |
| Positive Cluster |  |  |  |  |  |  |  |  |
| P1 | Left frontal pole Left middle frontal gyrus Left cerebral white matter adjacent to left frontal pole and middle frontal gyrus | .0007 | .4440 | < .001 | 219 | -32 | 32 | 18 |
| P2 | Right planum temporal | .0005 | .3456 | .003 | 175 | 47 | -34 | 15 |
| P3 | Right Heschl’s gyrus | .0004 | .3578 | .009 | 155 | 37 | -25 | 7 |
| P4 | Left precentral gyrus left superior longitudinal fasciculus adjacent to left precentral gyrus | .0004 | .3959 | .010 | 153 | -46 | 2 | 20 |
| P5 | Right inferior temporal gyrus | .0004 | .3762 | .014 | 147 | 57 | -33 | -10 |
| Negative Cluster |  |  |  |  |  |  |  |  |
| N1 | Left frontal medial cortex  Left forceps minor | -.0003 | .5958 | .049 | 125 | -17 | 32 | -14 |

**Supplementary Table 5.** Characteristics of clusters with an association between PD patients’ microstructural metrics and postoperative change in time spent with poor mobility (OFF). “Positive Cluster” denotes clusters with a positive association between patients’ microstructural metrics and postoperative changes in time spent in OFF, i.e. higher values of a specific metric were associated with higher postoperative values. “Negative Cluster” denotes clusters with a negative association between patients’ microstructural metrics and postoperative difference in time spent in OFF, i.e. higher values of a specific metric were associated with lower postoperative values. “Location” indicates the anatomical landmark comprising the majority of voxels of a cluster according to Johns Hopkins University (JHU) white matter atlas, Harvard-Oxford cortical and subcortical atlas, and University College London (UCL) cerebellar atlas. P-Values are clusterwise p-values corrected for multiple comparisons. “Volume in mm³” denotes the size of a cluster and “MNI152-coordinates” describes the coordinates of the cluster’s center of gravity in MNI152-space.

**Supplementary Table 6**

|  |  | Baseline | | 3-MFU | | | Baseline vs.  3-MFU |  | 12-MFU | | | Baseline vs.  12-MFU | |
| --- | --- | --- | --- | --- | --- | --- | --- | --- | --- | --- | --- | --- | --- |
|  | *n* | *M* | *SD* | *n* | *M* | *SD* | *p* |  | *n* | *M* | *SD* | | *p* |
| UPDRS total score | 34 | 40.7 | 14.7 | 23 | 32.0 | 12.3 | **.006** |  | 27 | 31.1 | 14.6 | | **.003** |
| UPDRS part I | 34 | 2.9 | 2.1 | 29 | 1.8 | 1.3 | **.035** |  | 34 | 2.1 | 1.7 | | .059 |
| UPDRS part II | 34 | 8.3 | 5.4 | 29 | 7.2 | 4.7 | .185 |  | 34 | 7.8 | 5.8 | | .288 |
| UPDRS part III | 35 | 24.9 | 11.9 | 23 | 19.2 | 8.3 | **.010** |  | 27 | 18.4 | 10.2 | | **.024** |
| UPDRS part IV | 34 | 6.0 | 3.1 | 29 | 3.9 | 3.0 | **.003** |  | 34 | 3.3 | 2.9 | | **<.001** |
| Movement Record |  |  |  |  |  |  |  |  |  |  |  | |  |
| ON [%] | 35 | 37.1 | 18.6 | 25 | 52.1 | 18.6 | **.018** |  | 30 | 55.7 | 12.7 | | **<.001** |
| OFF [%] | 35 | 23.9 | 15.9 | 25 | 13.5 | 16.7 | **.018** |  | 31 | 7.8 | 12.5 | | **<.001** |
| SLEEP [%] | 35 | 31.9 | 11.1 | 25 | 32.3 | 8.4 | .530 |  | 31 | 36.1 | 12.8 | | .102 |
| ON TD [%] | 35 | 7.0 | 12.65 | 25 | 2.1 | 3.9 | .057 |  | 31 | 2.1 | 4.9 | | .102 |
| PDQ-8 SI | 35 | 32.8 | 15.3 | 29 | 24.5 | 14.2 | **.002** |  | 33 | 23.6 | 15.2 | | **<.001** |
| LEDD [mg] | 35 | 964.9 | 408.3 | 29 | 614.4 | 311.8 | **<.001** |  | 32 | 541.5 | 296.5 | | **<.001** |
| LEDD DA [mg] | 35 | 266.5 | 130.7 | 29 | 216.1 | 202.2 | **.02** |  | 32 | 165.9 | 129.5 | | **<.001** |

**Supplementary Table 6:** Outcome parameters at baseline and 3 as well as 12-months follow-up. Values for movement records represent percentage values. Reported p-values are corrected for multiple comparisons using Benjamini-Hochberg’s method for eight scales. Bold font highlights significant results, p<.05.

**Abbreviations:** 3-MFU = 3-month follow-up; 12-MFU = 12-month follow-up; LEDD = Levodopa equivalent daily dose; LEDD-DA = LEDD of Dopamine Agonists; PDQ-8 SI = 8-item Parkinson’s Disease Questionnaire summary index; SD = standard deviation; UPDRS = Unified Parkinson’s Disease Rating Scale.

**Supplementary Figures**

**Supplementary Figure 1**


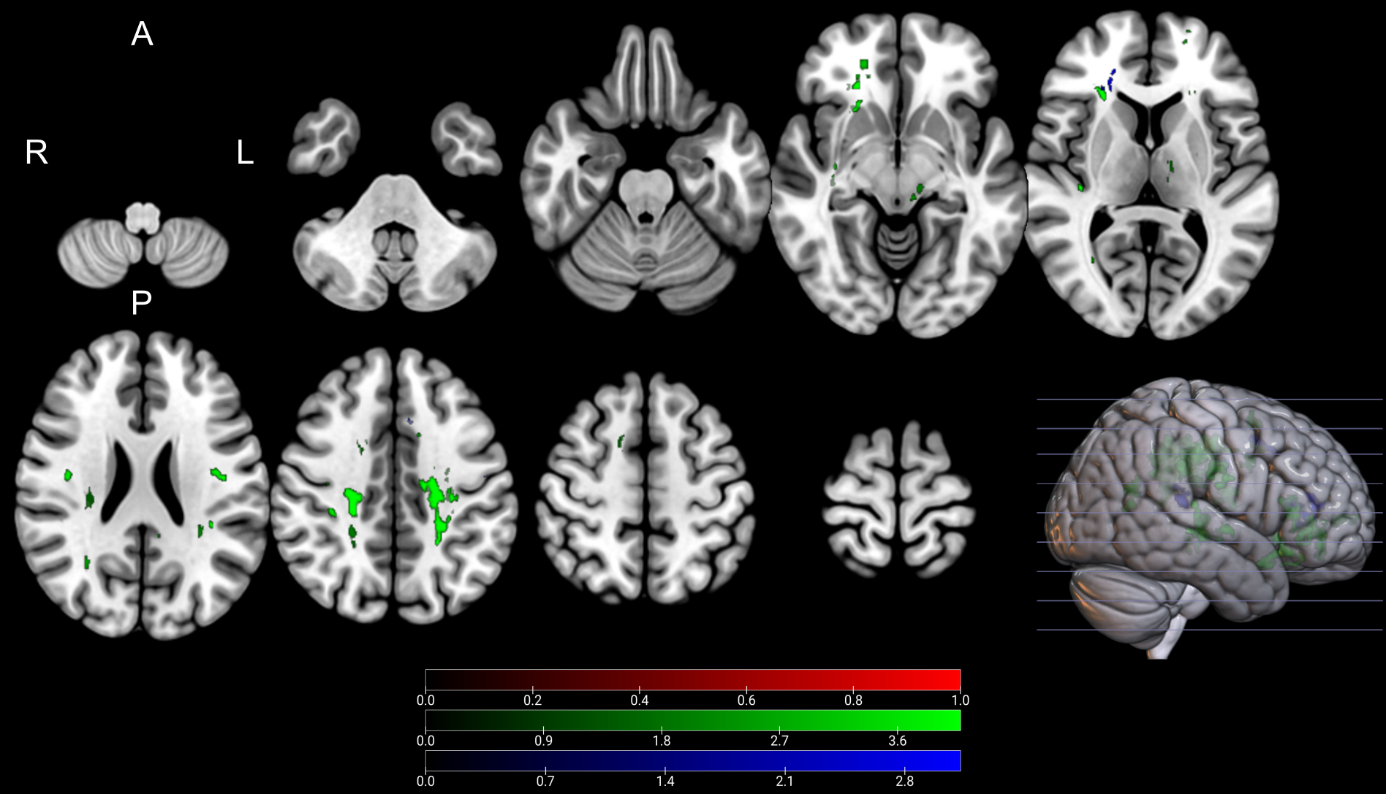


**Supplementary Figure 1.** Clusters with a positive association between PD patients’ FA-values and postoperative change in UPDRS (red), time spent in ON (green), and time spent in OFF (blue), as revealed by the whole brain analysis. P-Values were corrected for multiple comparisons using a permutation-based approach and shown as the negative decadic logarithm of the p-value.

**Supplementary Figure 2**

**
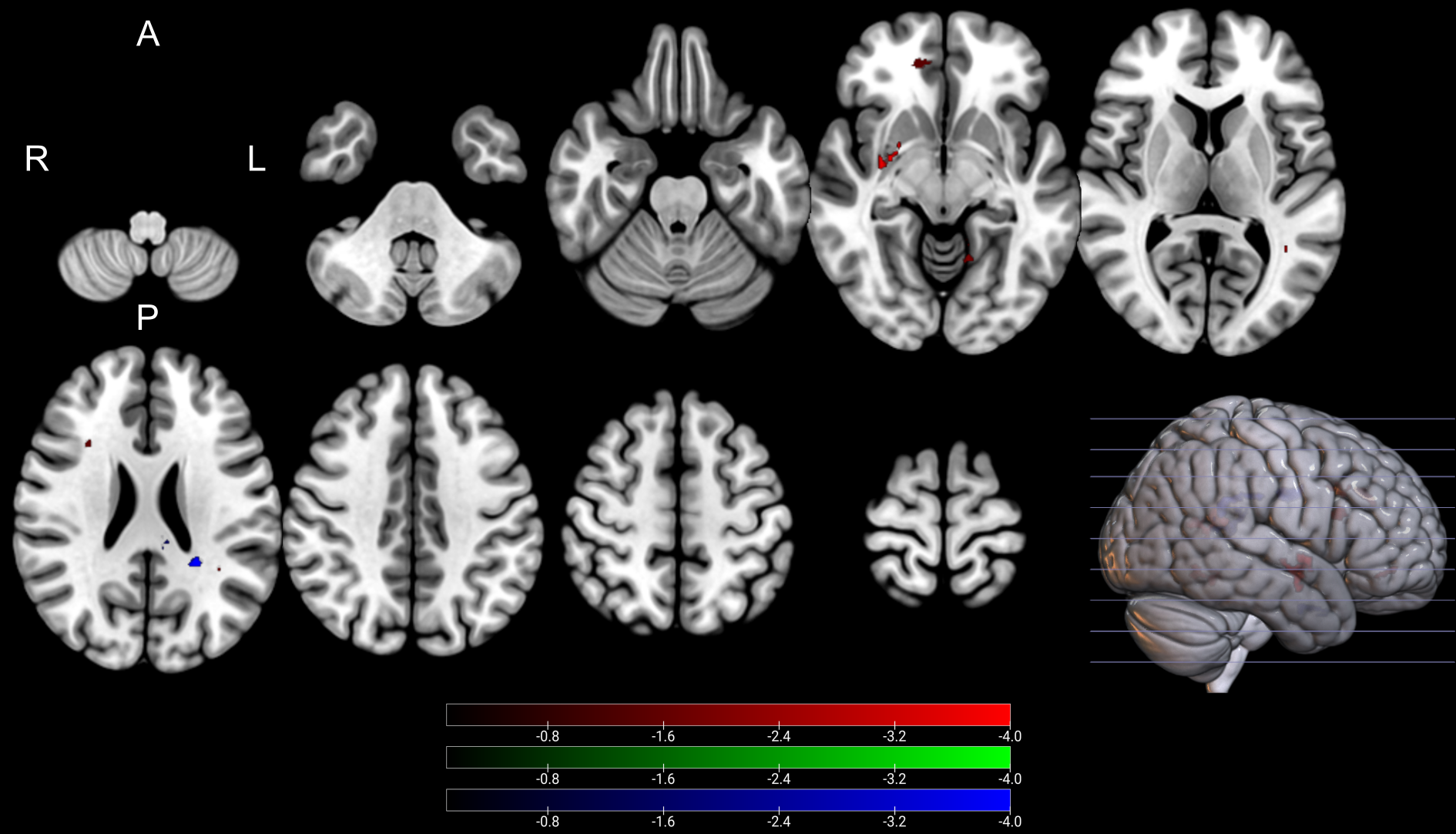
**

**Supplementary Figure 2.** Clusters with a negative association between PD patients’ FA-values and postoperative change in UPDRS (red), time spent in ON (green), and time spent in OFF (blue), as revealed by the whole brain analysis. P-Values were corrected for multiple comparisons using a permutation-based approach and shown as the negative decadic logarithm of the p-value.

**Supplementary Figure 3**


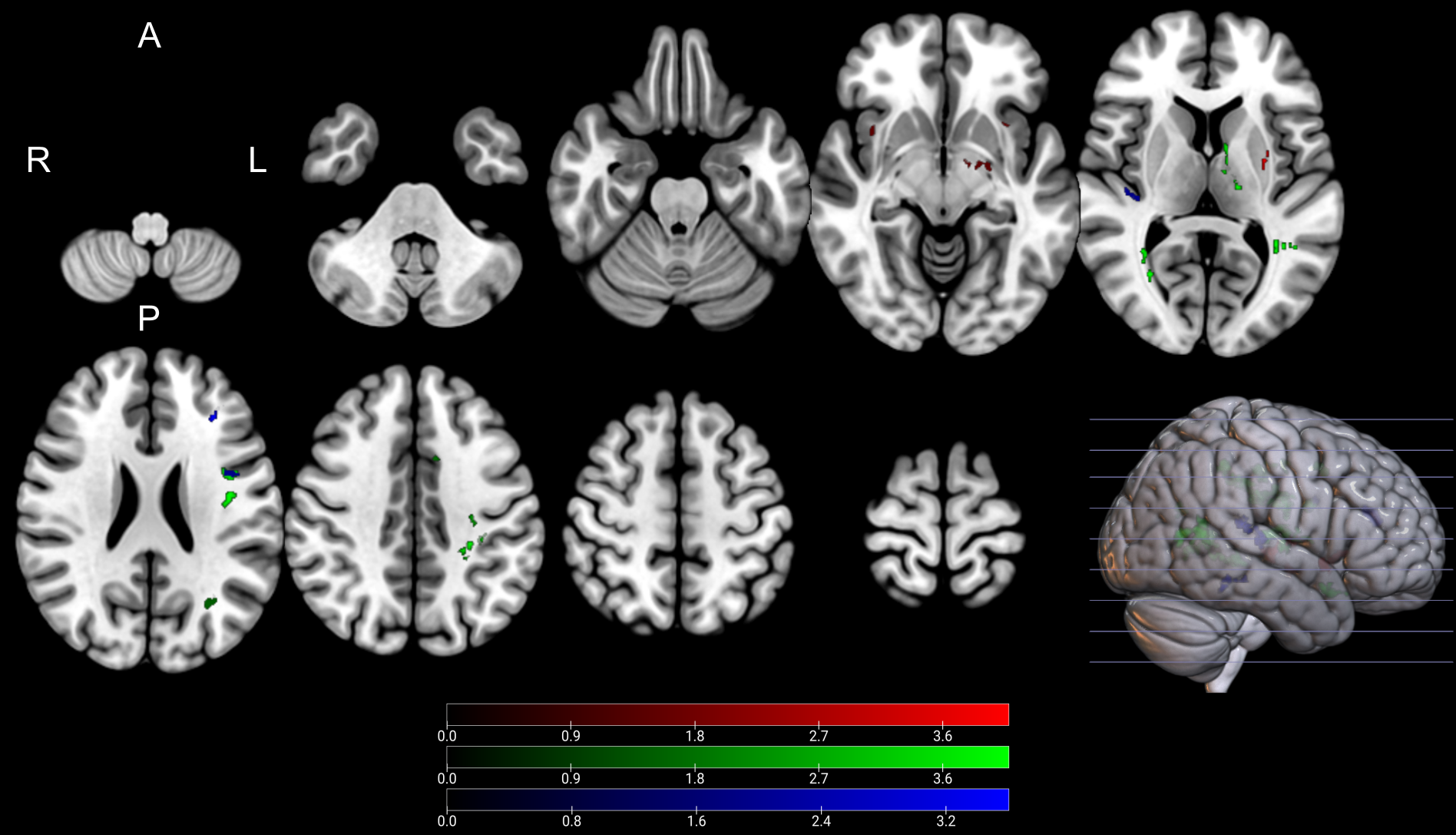


**Supplementary Figure 3.** Clusters with a positive association between PD patients’ NDI-values and postoperative change in UPDRS (red), time spent in ON (green), and time spent in OFF (blue), as revealed by the whole brain analysis. P-Values were corrected for multiple comparisons using a permutation-based approach and shown as the negative decadic logarithm of the p-value.

**Supplementary Figure 4**


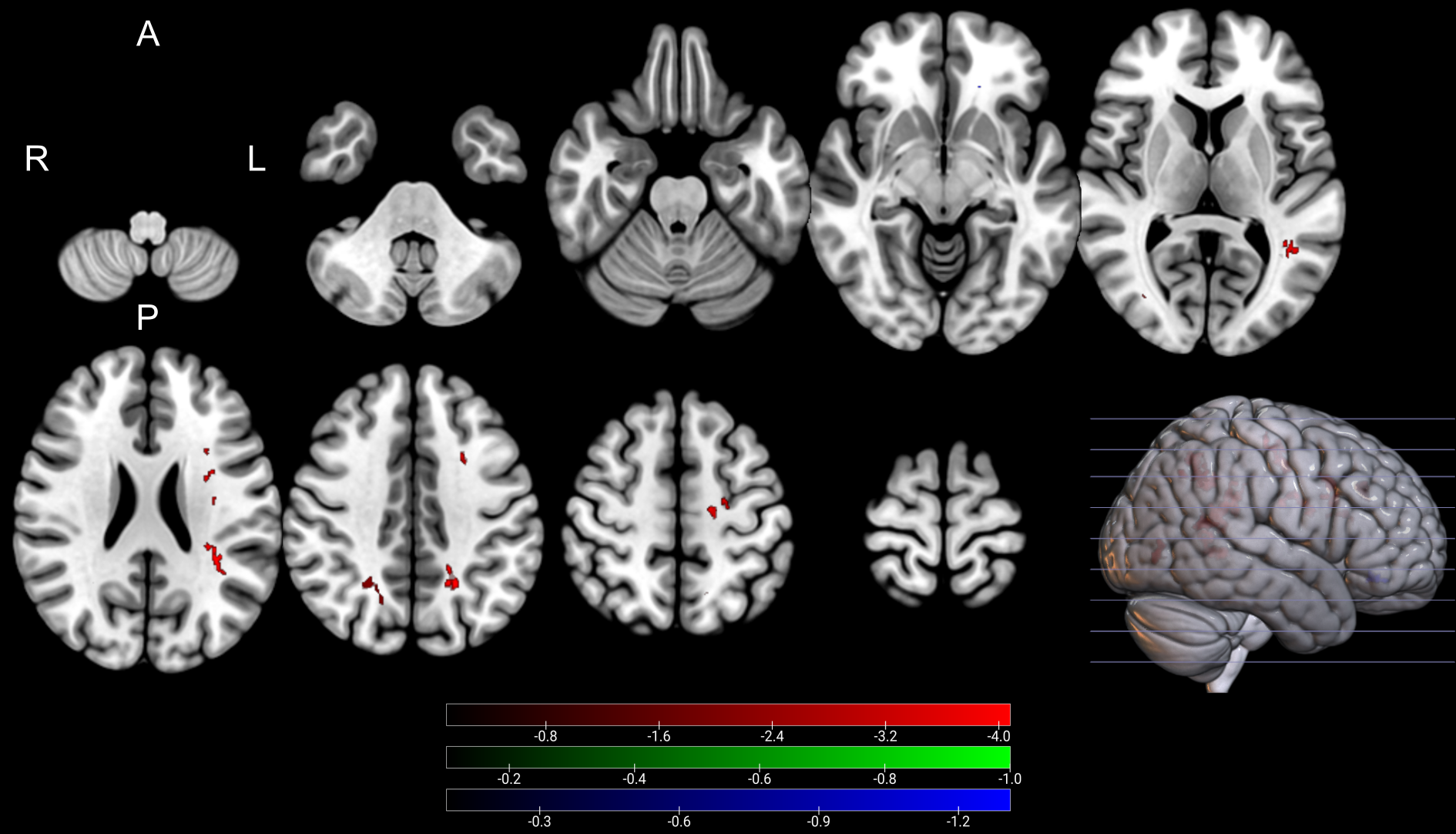


**Supplementary Figure 4.** Clusters with a negative association between PD patients’ NDI-values and postoperative change in UPDRS (red), time spent in ON (green), and time spent in OFF (blue), as revealed by the whole brain analysis. P-Values were corrected for multiple comparisons using a permutation-based approach and shown as the negative decadic logarithm of the p-value.

**Supplementary Figure 5**

**
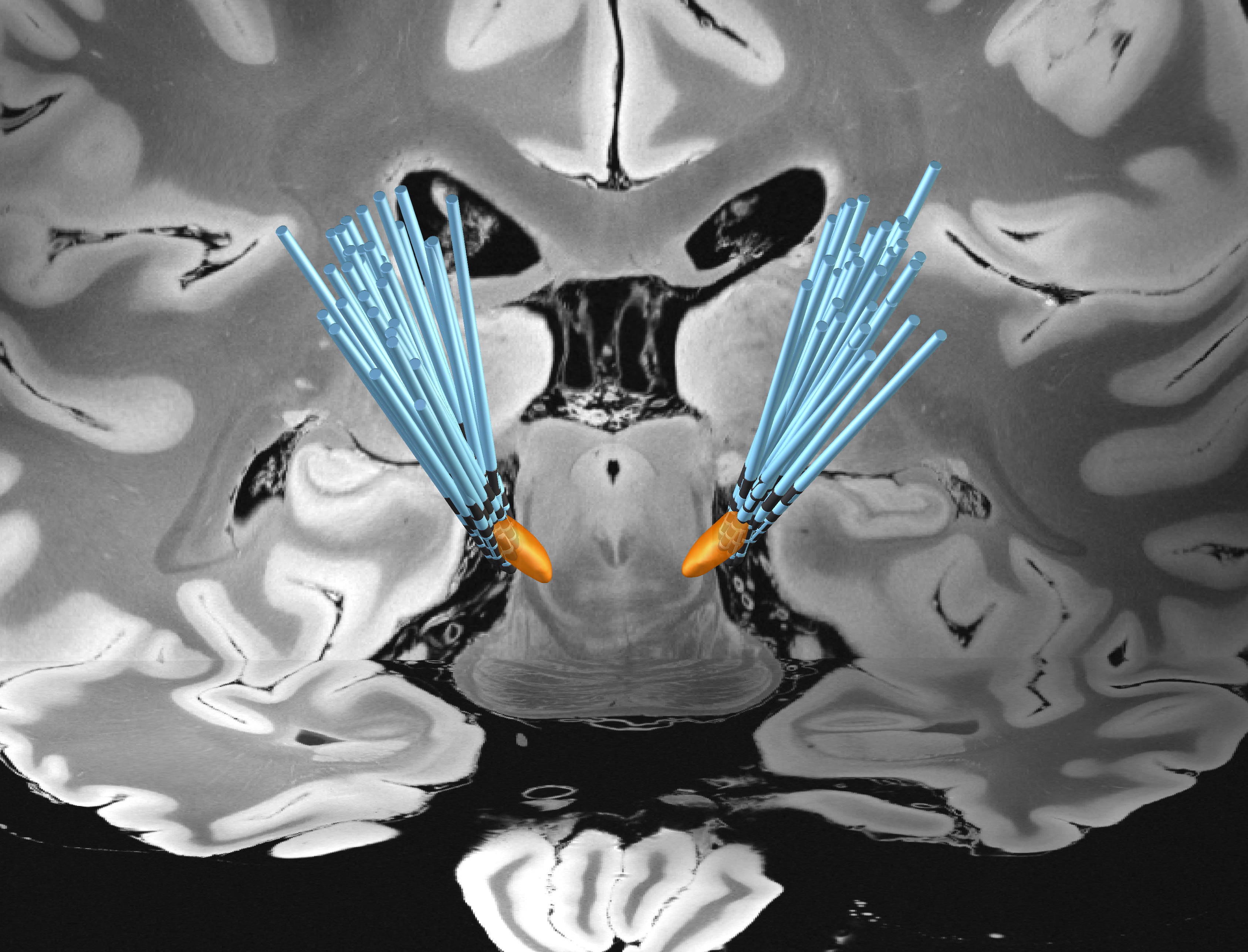
**

**Supplementary Figure 5** Reconstruction of the electrodes for the 35 patients as calculated within the Lead-DBS toolbox.

**Supplementary Figure 6**


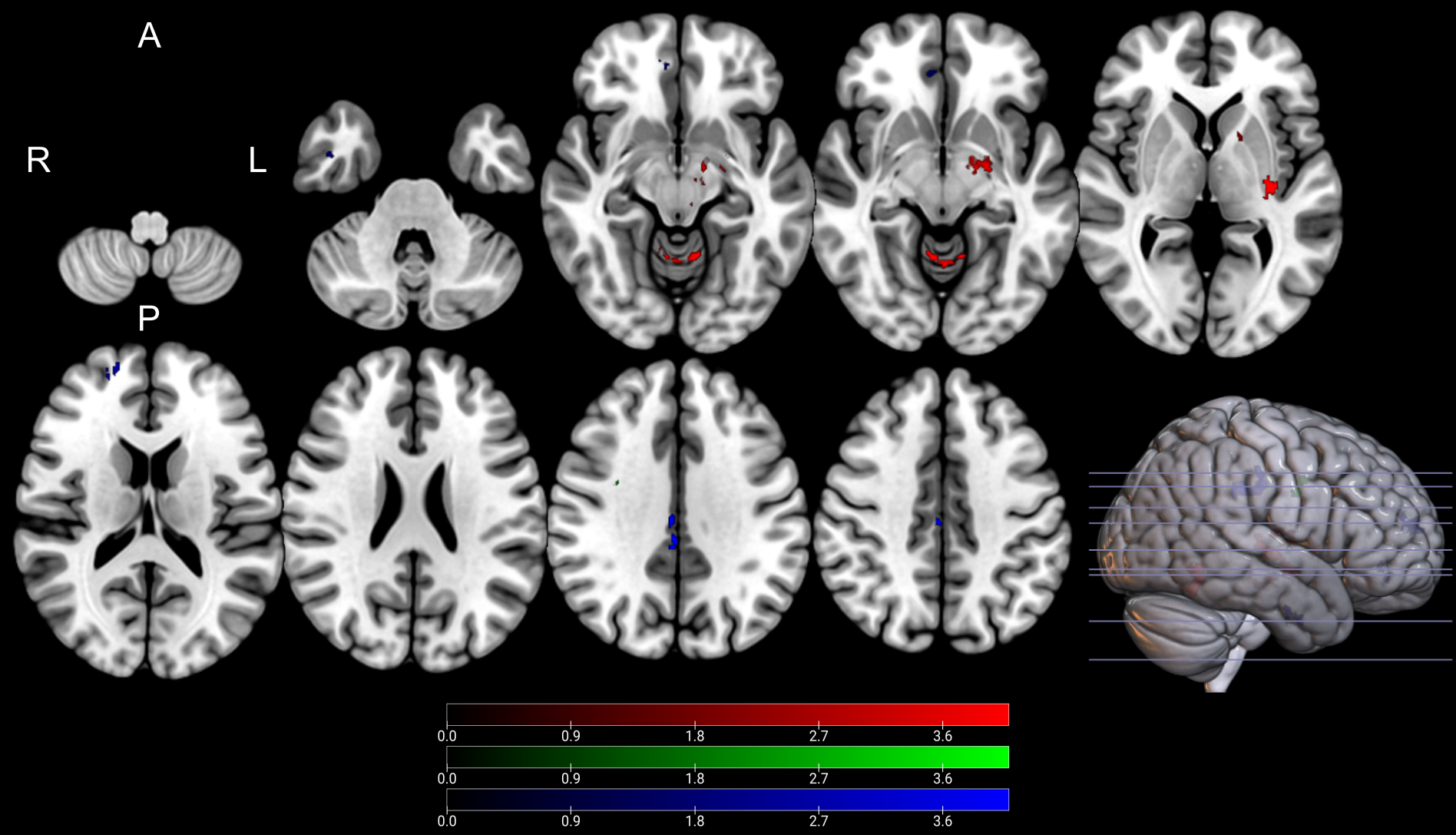


**Supplementary Figure 6.** Clusters with a positive association between PD patients’ ODI-values and postoperative change in UPDRS (red), time spent in ON (green), and time spent in OFF (blue), as revealed by the whole brain analysis, corrected for LEDD, age, and disease duration. Clusterwise P-Values were corrected for multiple comparisons using a permutation-based approach and shown as the negative decadic logarithm of the p-value.

**Supplementary Figure 7**


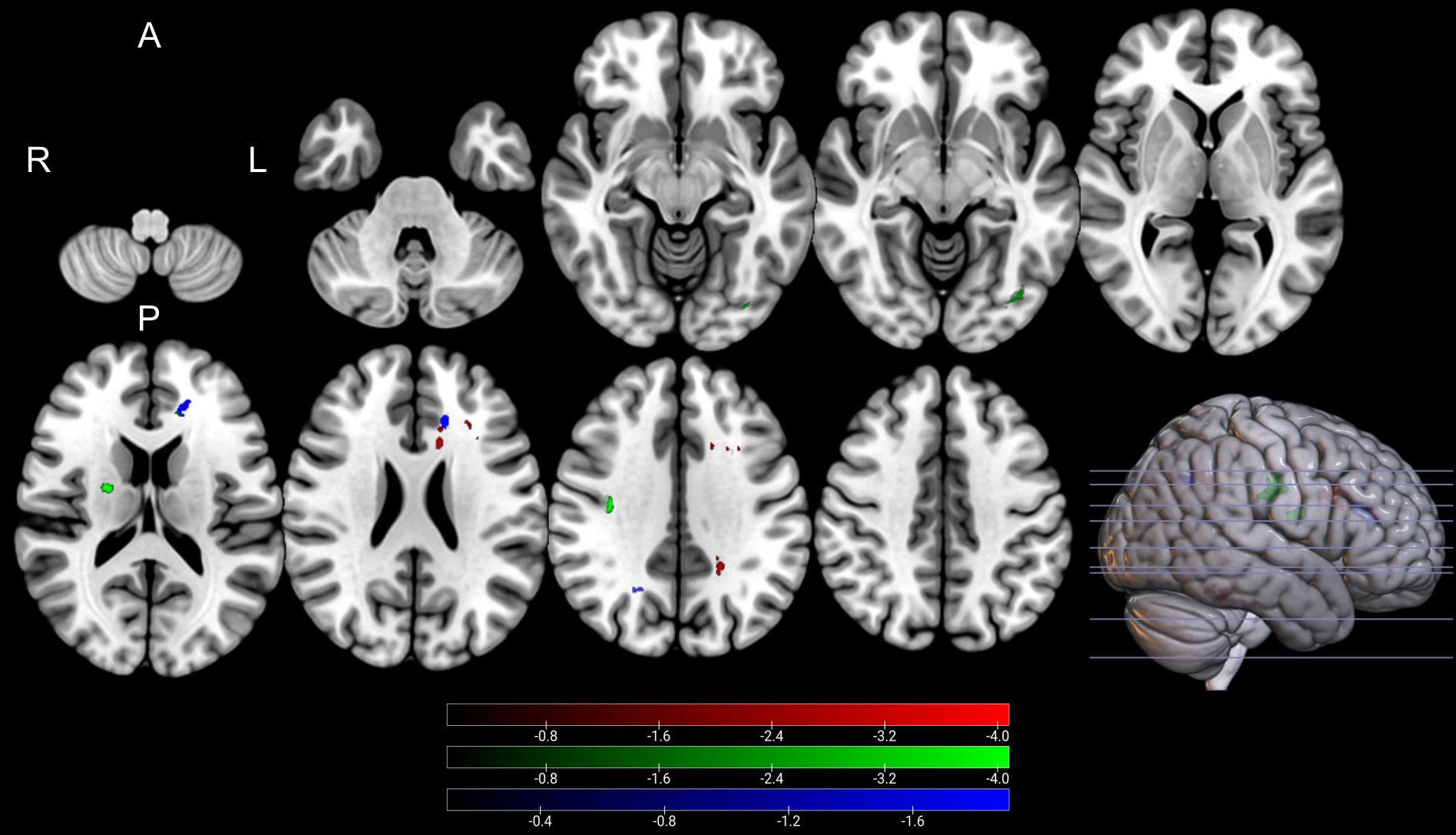


**Supplementary Figure 7.** Clusters with a negative association between PD patients’ ODI-values and postoperative change in UPDRS (red), time spent in ON (green), and time spent in OFF (blue), as revealed by the whole brain analysis, corrected for LEDD, age, and disease duration. P-Values were corrected for multiple comparisons using a permutation-based approach. Clusterwise P-Values were corrected for multiple comparisons using a permutation-based approach and shown as the negative decadic logarithm of the p-value.

**Supplementary Figure 8**


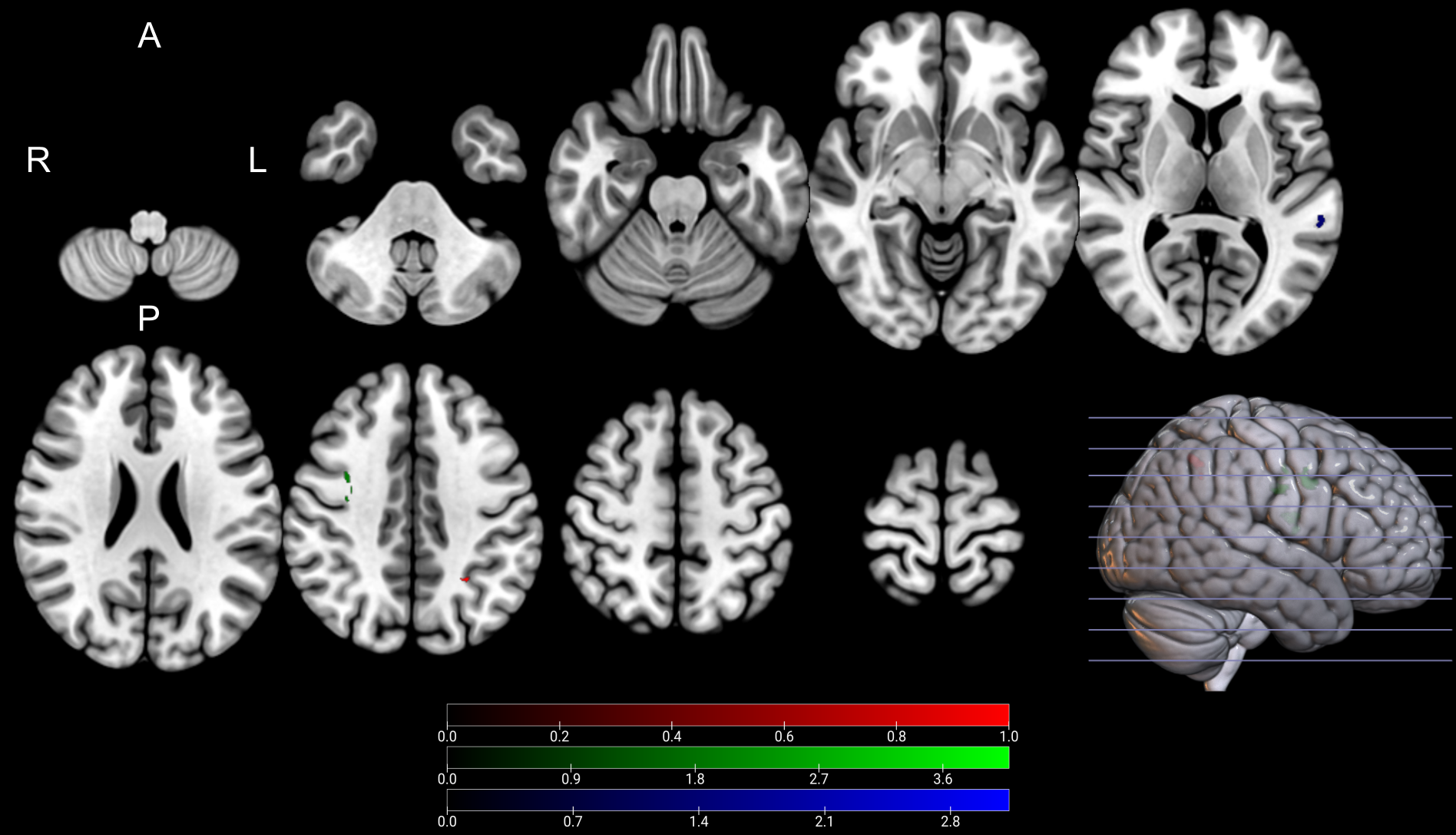


**Supplementary Figure 8.** Clusters with a positive association between PD patients’ FA-values and postoperative change in UPDRS (red), time spent in ON (green), and time spent in OFF (blue), as revealed by the whole brain analysis, corrected for LEDD, age, and disease duration. P-Values were corrected for multiple comparisons using a permutation-based approach and shown as the negative decadic logarithm of the p-value.

**Supplementary Figure 9**

**
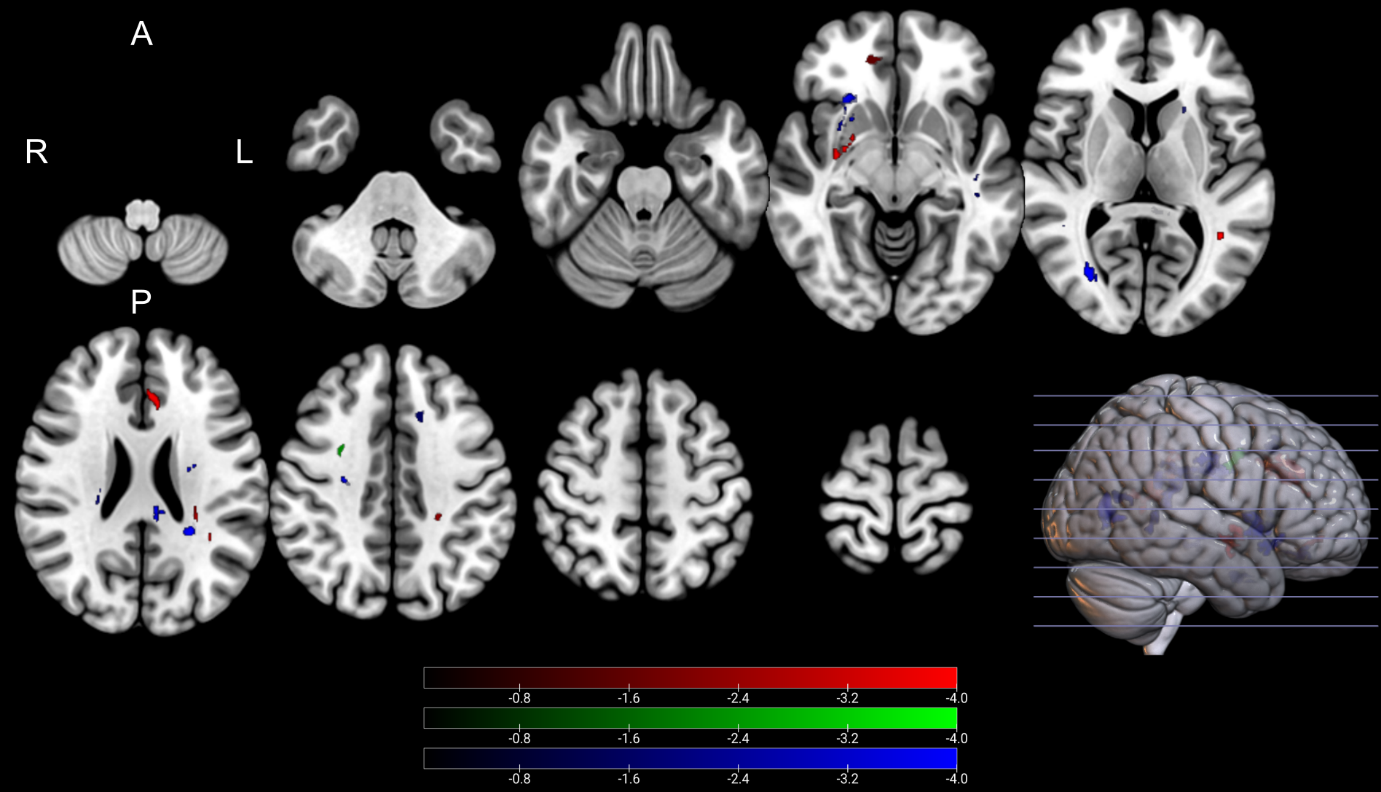
**

**Supplementary Figure 9.** Clusters with a negative association between PD patients’ FA-values and postoperative change in UPDRS (red), time spent in ON (green), and time spent in OFF (blue), as revealed by the whole brain analysis, corrected for LEDD, age, and disease duration. P-Values were corrected for multiple comparisons using a permutation-based approach and shown as the negative decadic logarithm of the p-value.

**Supplementary Figure 10**

**
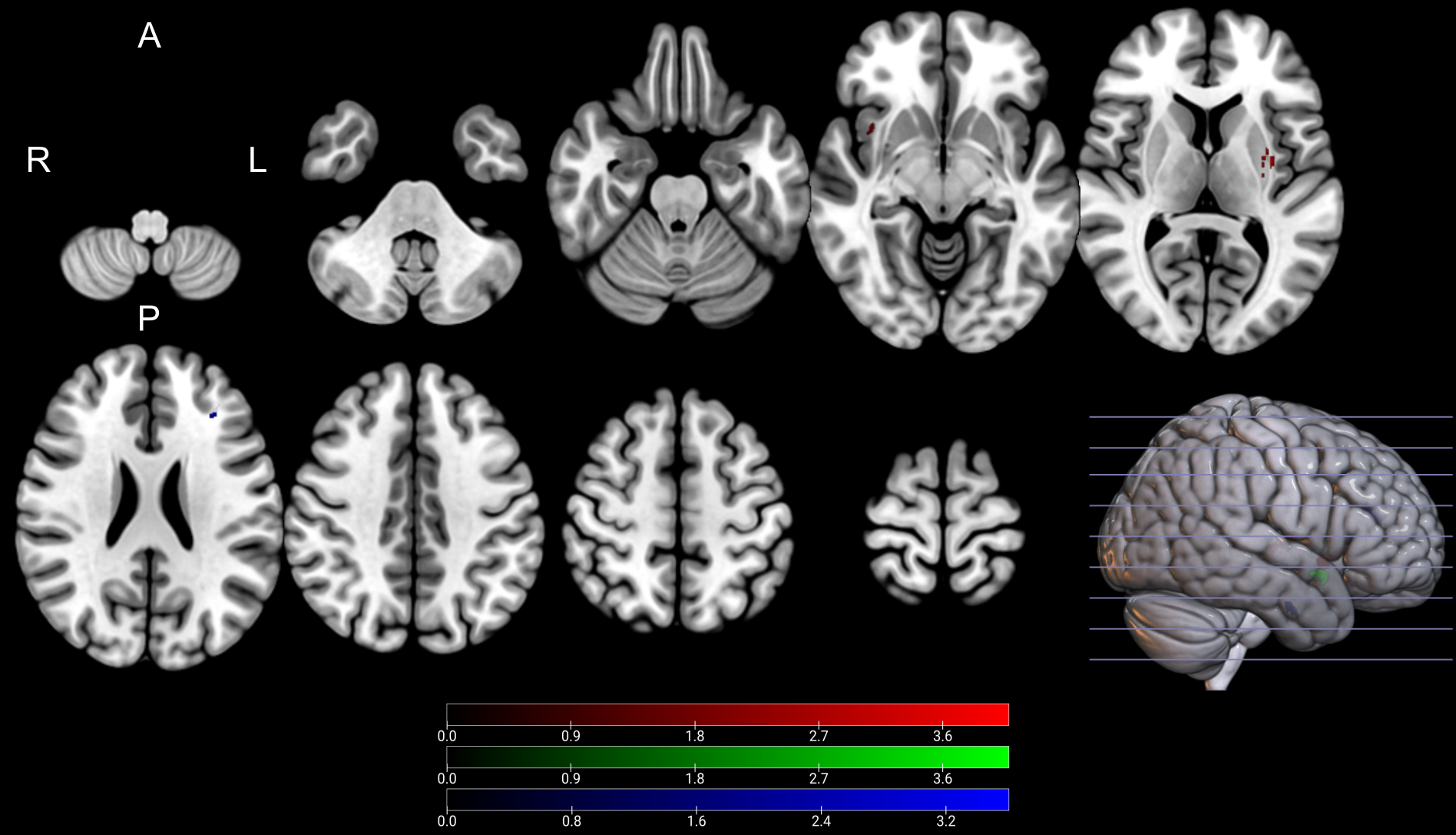
**

**Supplementary Figure 10.** Clusters with a positive association between PD patients’ NDI-values and postoperative change in UPDRS (red), time spent in ON (green), and time spent in OFF (blue), as revealed by the whole brain analysis, corrected for LEDD, age, and disease duration. P-Values were corrected for multiple comparisons using a permutation-based approach and shown as the negative decadic logarithm of the p-value.

**Supplementary Figure 11**


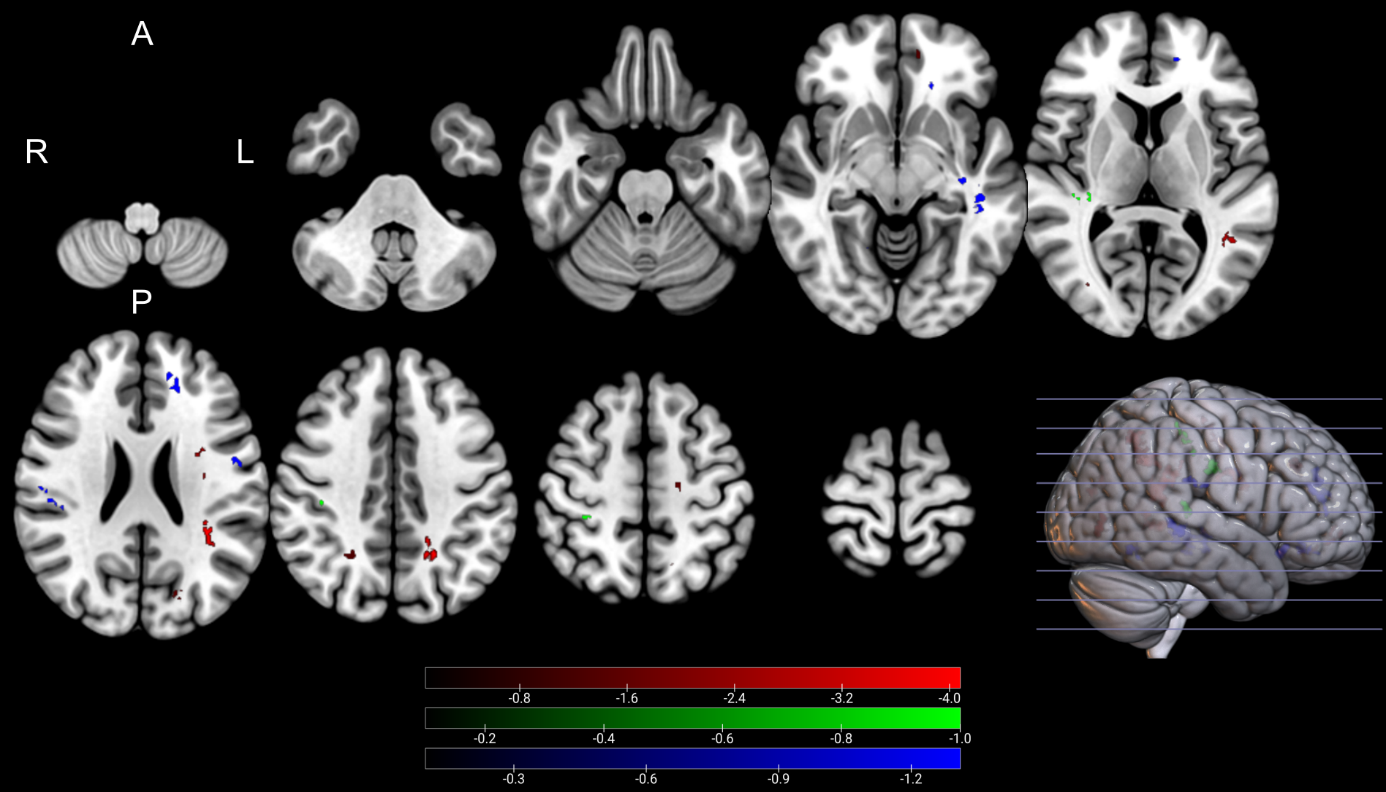


**Supplementary Figure 11.** Clusters with a negative association between PD patients’ NDI-values and postoperative change in UPDRS (red), time spent in ON (green), and time spent in OFF (blue), as revealed by the whole brain analysis, corrected for LEDD, age, and disease duration. P-Values were corrected for multiple comparisons using a permutation-based approach and shown as the negative decadic logarithm of the p-value.
